# Trimesic Acid as a Building Block for Ternary and Quaternary Salts and Salt Cocrystals

**DOI:** 10.1021/acs.cgd.4c00779

**Published:** 2024-10-31

**Authors:** Lamis Alaa Eldin Refat, Marwah Aljohani, Andrea Erxleben

**Affiliations:** † School of Biological and Chemical Sciences, University of Galway, Galway H91TK33, Ireland; ‡ Synthesis and Solid State Pharmaceutical Centre (SSPC), Limerick V94 T9PX, Ireland; § Department of Chemistry, College of Science, Imam Abdulrahman Bin Faisal University, P.O. Box 76971, Dammam 31441, Saudi Arabia

## Abstract

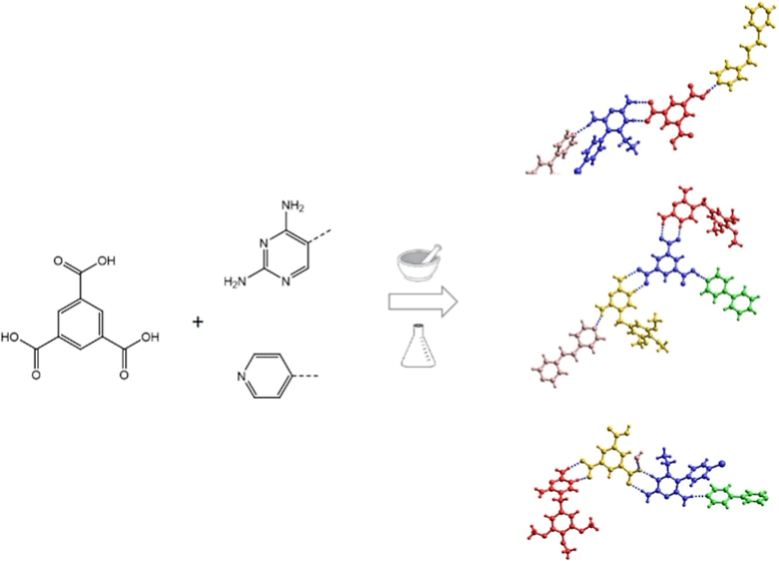

In the field of cocrystals, the synthon-based design of two-component crystals is well established and the interest is now shifting toward higher order cocrystals as the next challenge. Carboxylic acids form a robust synthon with pyridyl coformers and interact with 2-aminopyrimidines through a pair of strong, charge-assisted hydrogen bonds. In this work we describe the formation of higher order salts and salt cocrystals of trimesic acid using 2,4-diaminopyrimidine (pyrimethamine, trimethoprim) and pyridyl (4,4′-bipyridine, 1,2-di(4-pyridyl)ethylene, 1,3-di(4-pyridyl)propane, 4-phenylpyridine) coformers. We report the single crystal structures of five binary, eight ternary and three quaternary systems with the compositions AB, A_2_B, A_3_B, A_2_BC, A_3_BC, A_2_BC_1.5_, ABC_1.5_, A^1^_2_A^2^B, ABC^1^C,^2^ A_2_BC^1^C^2^_0.5_, and A^1^A^2^BC where A is a 2,4-diaminopyrimidine cation, B is the mono-, di- or trianion of trimesic acid and C is a pyridine. We also show that the ternary and quaternary salts and salt cocrystals can be prepared in bulk quantities by liquid-assisted grinding.

## Abstract 2

Ternary and quaternary cocrystals of trimesic acid with pyridyl and 2,4-diaminopyrimidine coformers have been obtained by solution crystallization and liquid-assisted grinding.

## Introduction

Multicomponent crystals not only present important materials with enhanced properties and functions, but also continue to fascinate the crystal engineer by their aesthetic appeal and the intellectual challenge of their rational synthesis.^1^ The latter is particularly true for three-component and higher-order cocrystals. The intermolecular interactions and the relative solubilities of the coformers and potential combinations of binary systems need to be carefully balanced in order to obtain multicomponent cocrystals. A solvent has to be found in which the individual components have a similar solubility. While this can already be problematic, the low solubility of possible lower-order cocrystals can present an even bigger hurdle. While many cocrystals have been identified through systematic and comprehensive screening studies, the need for robust and reliable supramolecular reactions to allow a more predictive approach has been emphasized in the literature.^1−3^ One supramolecular design strategy of ternary cocrystals uses a ditopic, unsymmetric coformer that can form two distinct synthons or hydrogen bonds of different strength.^2,4,5^ This approach is based on Etter’s rule that states that the best hydrogen bond donor preferentially interacts with the best hydrogen bond acceptor, while the second-best hydrogen bond donor connects to the second-best acceptor and so on.^6^ The first cocrystal that was built exploiting hierarchical intermolecular interactions was reported by Aakeröy and co-workers who attached a stronger and a weaker acid to the pyridine and amide moieties of isonicotinamide, respectively.^2^ Jain et al. exploited synthon hierarchy and packing mimicry to obtain ternary cocrystals solely based on halogen bonding.^7^ Other design strategies combine complementary intermolecular interactions, in particular hydrogen bonding and halogen bonding.^8−11^ Seaton et al.^12^ and Lemmerer and co-workers^13,14^ designed ternary cocrystals by combining hydrogen bonding and charge transfer interactions. Chakraborty et al. used π–π stacking and weak hydrogen bonding.^15^ Adsmond et al. obtained ternary cocrystals by cocrystallizing carboxyphenols with two complementary acceptors.^16^ In a host–guest design a third component is accommodated into the voids of a binary system.^17−21^ Examples are the inclusion of 1,4-dioxane and other guest molecules into a caffeine-succinic acid host^17^ and inclusion compounds based on calix[4]arene-pyridine assemblies.^18^ Tetraarylpyrene forms a host lattice with three distinct inclusion sites and two different guest molecules have been fitted simultaneously into different voids.^22^

Desiraju and co-workers showed that a ternary cocrystal can be obtained when a coformer occupies two crystallographic sites in a binary system and one is loosely bound and easily substituted with a molecule of similar size and shape.^23^ This strategy was first applied to 1,3,5,7-tetrabromoadamantane/urotropine/CBr_4_^23^ and later to 2- and 5-methylresorcinol, bipyridine and mimics of bipyridine to confirm the generality of the approach.^24^ Smolka et al. prepared a 2:3 binary cocrystal of 2,2-dihydroxybiphenyl and phenazine that contained two types of phenazine, one forming two hydrogen bonds and one forming only one hydrogen bond. The latter could be replaced by an acridine molecule to give a three-component crystal.^25^

More recently the structural inequivalence/shape-size mimicry strategies have been extended to quaternary^26^ and even (nonstoichiometric) five- and six-component^27−29^ cocrystals. Mir et al. combined the structural inequivalence strategy with the long-range synthon Aufbau module (LSAM) concept^30,31^ to synthesize ternary and quaternary cocrystals based on resorcinol derivatives.^31,32^ Rajkumar reported quaternary cocrystals of 3,5-dihydroxybenzoic acid using the LSAM approach and hierarchical hydrogen bonding interactions^33^ and Ahsan and Mukherjee obtained ternary and quaternary cocrystals of 2,7-dihydroxynaphthalene with large modular synthons.^34^ In these and other examples,^35^ the higher order cocrystal is built from a symmetric multitopic ligand (resorcinol, 2,3-dihydroxybenzoic acid, 2,7-dihydroxynaphthalene). The symmetric tritopic ligand 1,3,5-cyclohexanetricarboxylic acid has also been used as a building block for ternary cocrystals. Nangia and co-workers prepared ternary cocrystals by combining this tricarboxylic acid with two bipyridyl coformers with different flexibility or basicity.^36−38^ Several of the structures contained voids that could accommodate a fourth component.^36,38^

**Figure 1:**
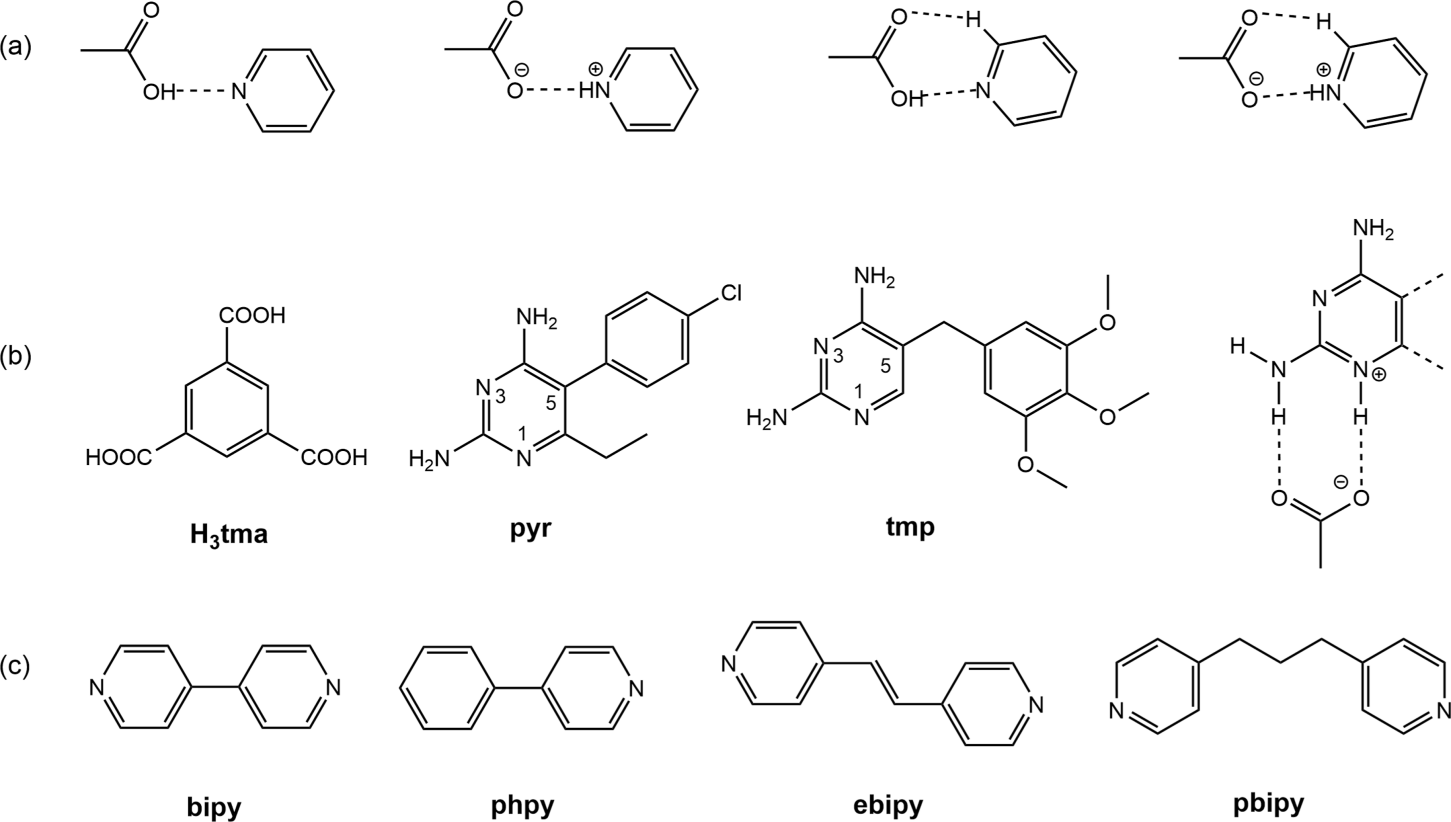
(a) Synthons between carboxylic acids and pyridines. (b) Chemical structures of H_3_tma, pyr and tmp and *R*_2_^2^(**8**) motif with charge-assisted hydrogen bonding between the C2–NH_2_,N_pyrimidine_ site and a carboxylic acid. (c) Pyridine coformers used in this study.

In this work we have used the aromatic tricarboxylic acid trimesic acid (H_3_tma) to construct ternary and quaternary cocrystals by solution crystallization as well as liquid-assisted grinding. Liquid-assisted grinding is generally considered an advantageous preparation method for cocrystals due to its “green” nature and quantitative yields.^39^ A number of binary cocrystals of H_3_tma with pyridyl,^40−49^ amine,^50,51^ azole,^52^ purine,^53,54^ pyrimidine,^55,56^ melamine,^57^ and amide^58−61^ coformers have been reported in the literature. The carboxylic acid–pyridine synthon is one of the most frequently used synthons in cocrystal design.^62^ Different variations of this synthon are shown in Figure 1a. 2-Aminopyridines and carboxylic acids typically form the robust *R*_2_^2^(**8**) motif with a pair of NH_2_···O=C and N_pyridine_···HO–C/NH^+^_pyridine_···^–^O–C hydrogen bonds. Previous work by us^63,64^ and others^65−70^ showed that this hydrogen bonding motif is the main synthon in pyrimethamine (pyr) and trimethoprim (tmp) carboxylates that both contain an *ortho*-NH_2_,N_pyrimidine_ binding site as well as additional hydrogen bond donor and acceptor functionalities (Figure 1b). We reasoned that by carefully controlling the stoichiometric ratios it should be possible to “decorate” a H_3_tma molecule (or its mono-, di- or trianion) with tmp, pyr and pyridines, thus designing higher-order salt cocrystals. We therefore performed a systematic cocrystallization study of H_3_tma with different combinations of tmp, pyr and the pyridines 4,4′-bipyridine (bipy), 1,2-di(4-pyridyl)ethylene (ebipy), 1,3-di(4-pyridyl)propane (pbipy), and 4-phenylpyridine (phpy).

## Experimental Section

### Materials

Pyr, tmp, H_3_tma, bipy, ebipy, pbipy, and phpy were purchased from Tokyo Chemical Industry Europe. The solvents acetonitrile, methanol (Merck Millipore), ethanol (Fisher) and dimethylformamide (Sigma-Aldrich) were analytical grade and were used as received.

### Solution Crystallization

Twenty mg of tmp or pyr and stoichiometric equivalents of the other components were dissolved in a minimum amount of solvent (10–15 mL) in 20 mL glass vials. The vials were covered with parafilm with small holes to allow crystals to grow by slow evaporation. Crystals of Hpyr^+^H_2_tma^–^·H_2_O grew from ethanol. Crystals of Htmp^+^H_2_tma^–^·3H_2_O, (Hpyr^+^)_2_Htma^2–^·3H_2_O, (Htmp^+^)_2_Htma^2–^·5H_2_O, Hpyr^+^Htmp^+^Htma^2–^, (Hpyr^+^)_2_Htmp^+^tma^3–^·2CH_3_OH·2H_2_O, (Hpyr^+^)_2_Htma^2–^·ebipy·H_2_O·CH_3_OH, (Htmp^+^)_2_Htma^2–^·1.5bipy·4H_2_O, (Htmp^+^)_3_tma^3–^·pbipy·7H_2_O, (Hpyr^+^)_2_Htma^2–^·bipy·H_2_O, Hpyr^+^Htmp^+^Htma^2–^·bipy·H_2_O, and Hpyr^+^H_2_tma^–^·phpy·pbipy grew from methanol. A mixture of methanol and acetonitrile resulted in crystals of (Hpyr^+^)_3_tma^3–^·CH_3_CN·CH_3_OH·2.5H_2_O, (Htmp^+^)_2_Htma^2–^·1.5ebipy·3H_2_O, and (Htmp^+^)_2_Htma^2–^·phpy·0.5ebipy·4H_2_O. Finally, a crystallization attempt with dimethylformamide resulted in the formation of crystals of Htmp^+^H_2_tma^–^·1.5bipy·H_2_O after leaving the vials uncovered inside a fumehood. Details of the crystallization experiments are provided in the Supporting Information (Table S1).

### Ball-Milling

Stoichiometric mixtures of tmp and/or pyr and the other components (Table S2) were placed in a 25 mL stainless steel milling jar along with 25–50 μL of solvent and a 15 mm diameter stainless steel ball. The samples were milled in an oscillatory ball mill (Mixer Mill MM400, Retsch GmbH & Co., Germany) at 25 Hz for 30 min.

### Thermal Analysis

Differential scanning calorimetry (DSC) plots were recorded in the 20 to 400 °C range using a PerkinElmer DSC 4000. The samples were heated in closed aluminum crucibles at a rate of 10 °C/min. Thermogravimetric analysis (TGA) plots were recorded using a PerkinElmer TGA 4000 in the range of 30 to 380 °C at a rate of 10 °C/min.

### X-ray Powder Diffraction

X-ray powder patterns of samples obtained by mechanical grinding and crystallization from solution were recorded on a Rigaku model Ultima IV diffractometer equipped with a Cu Kα radiation source. Data were acquired over the 2θ range between 5 and 50° (λ = 1.54178 Å, 40 kV, 40 mA).

### Single Crystal X-ray Analysis

Single crystal X-ray data of Hpyr^+^H_2_tma^–^·H_2_O (**1**), Htmp^+^H_2_tma^–^·3H_2_O (**2**), (Hpyr^+^)_2_Htma^2–^·3H_2_O (**3**), (Hpyr^+^)_3_tma^3–^·CH_3_CN·CH_3_OH·2.5H_2_O (**4**), (Htmp^+^)_2_Htma^2–^·5H_2_O (**5**), (Hpyr^+^)_2_Htma^2–^·ebipy·H_2_O·CH_3_OH (**6**), (Htmp^+^)_2_Htma^2–^·1.5bipy·4H_2_O (**7**), (Htmp^+^)_3_tma^3–^·pbipy·7H_2_O (**8**), (Hpyr^+^)_2_Htma^2–^·bipy·H_2_O (**9**), Htmp^+^H_2_tma^–^·1.5bipy·H_2_O (**10**), (Htmp^+^)_2_Htma^2–^·1.5ebipy·3H_2_O (**11**), Hpyr^+^Htmp^+^Htma^2–^ (**12**), (Hpyr^+^)_2_Htmp^+^tma^3–^·2CH_3_OH·2H_2_O (**13**), Hpyr^+^H_2_tma^–^·phpy·pbipy (**14**), (Htmp^+^)_2_Htma^2–^·phpy·0.5ebipy·4H_2_O (**15**), and Hpyr^+^Htmp^+^Htma^2–^·bipy·H_2_O (**16**) were collected on an Oxford Diffraction Xcalibur diffractometer. The X-ray structures were solved by direct methods using SHELXT and refined using SHELXL 2018/3 implemented in the Oscail package.^71−73^ The N–H and C–OH hydrogens of **2**, **4**, **6**, **9–12**, **14**, and **16** were located in the difference Fourier maps and refined isotropically. For **1** and **15** the N–H and C–OH hydrogens could not be found and were generated geometrically. In the case of **3**, **5**, **7**, **8**, and **13**, the treatment of the N–H and C–OH hydrogens was mixed with some positions being obtained from the difference Fourier maps and some positions being determined geometrically. Hydrogen atoms of all or some water molecules of crystallization of **1–5**, **7–9**, **11**, **13**, and **15** could not be located/generated geometrically. All C–H hydrogen atoms were placed in calculated positions and refined as riding atoms. Some of the water molecules of crystallization in **1–5**, **7**, and **13** have partial occupancies and are disordered. Disorder is also observed for the ebipy and bipy molecules in **6**, **10**, **11**, and **15** as well as for one or more tmp methoxy groups in **8**, **11**, and **15**. Crystallographic data and details of the structure refinement can be found in the Supporting Information (Tables S3–S5). The cif files can be obtained free of charge at http://www.ccdc.cam.ac.uk/conts/retrieving.html or from the Cambridge Crystallographic Data Centre, Cambridge, UK with the REF codes 2360743–2360758.

## Results and Discussion

### Binary Salts of H_3_tma with Pyr and Tmp

We started our study by investigating if we can control the stoichiometry of binary H_3_tma-diaminopyrimidine salts. The 1:1 salts Hpyr^+^H_2_tma^–^·H_2_O (**1**) and Htmp^+^H_2_tma^–^·3H_2_O (**2**) crystallized from equimolar mixtures. Solutions containing pyr and H_3_tma in a 2:1 and 3:1 ratio gave (Hpyr^+^)_2_Htma^2–^·3H_2_O (**3**) and (Hpyr^+^)_3_tma^3–^·CH_3_CN·CH_3_OH·2.5H_2_O (**4**), respectively. The 2:1 mixture of tmp and H_3_tma formed very thin needles that were unsuitable for single crystal X-ray analysis. The 2:1 salt (Htmp^+^)_2_Htma^2–^·5H_2_O (**5**) was obtained from the solution of a 3:1 mixture of tmp and H_3_tma. X-ray powder diffraction (XRPD) analysis showed that **3** and **4** can also be prepared by solvent-drop milling in the presence of traces of methanol/water and methanol/water/acetonitrile, respectively (Figures S1 and S2). A comparison of the experimental and simulated patterns confirmed that the conversion of the physical mixture of H_3_tma and three equivalents pyr to (Hpyr^+^)_3_tma^3–^·CH_3_CN·CH_3_OH·2.5H_2_O is quantitative. By contrast, an additional peak was observed in the experimental pattern of the milled 2:1 sample indicating that the product was not phase pure. Milling 1:1, 2:1 and 3:1 mixtures of tmp and H_3_tma in the presence of solvent resulted in XRPD patterns with an amorphous halo, while the XRPD pattern of a milled 1:1 mixture of pyr and H_3_tma showed broad peaks of the salt with an underlying amorphous halo (Figures S3–S6).

**Figure 2:**
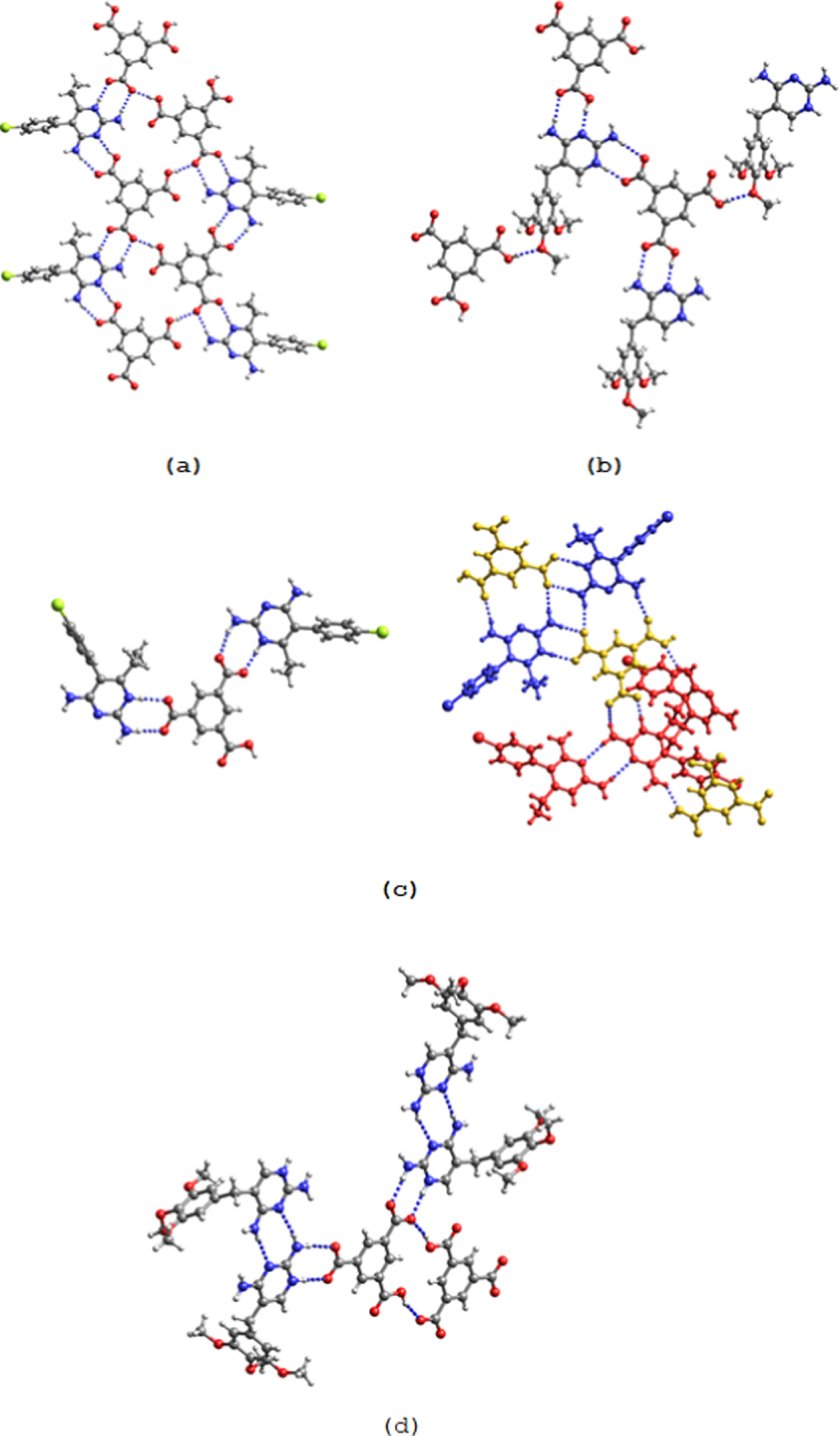
Crystal structures of (a) Hpyr^+^H_2_tma^–^·H_2_O (**1**), (b) Htmp^+^H_2_tma^–^·3H_2_O (**2**), (c) (Hpyr^+^)_2_Htma^2–^·3H_2_O (**3**) (blue: Hpyr^+^ A; red Hpyr^+^ B) and (d) (Htmp^+^)_2_Htma^2–^·5H_2_O (**5**). For clarity, the water molecules of crystallization are not shown.

The crystal structures of the five binary salts are shown in Figures 2 and S7. In all cases the C–O bond distances are equal within the standard deviations (Table S6) indicating that proton transfer from the carboxyl group to the N1 site had taken place so that the two components interact through charge-assisted hydrogen bonding with each other. The p*K*_a_ value of the N1 nitrogen of tmp is 7.16, while the p*K*_a1_, p*K*_a2_ and p*K*_a3_ values of H_3_tma are 2.12, 4.10 and 5.18, respectively. Proton transfer for the 1:1 composition is thus in line with the Δp*K*_a_ rule according to which a salt is obtained when the difference in the p*K*_a_ values of the coformers is > 4, whereas a Δp*K*_a_ of < −1 leads to a cocrystal.^74^ For the binding of a second and third tmpH^+^ cation, the p*K*_a_ differences are 3.16 and 1.98, respectively and thus fall in the salt-cocrystal continuum region where a prediction is not possible. Using the model developed by Cruz–Cabeza that estimates the probability for salt formation as
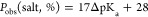
the probabilities of the second and third proton transfer are 82 and 62%.^74^

The asymmetric unit of the 1:1 pyr salt contains an N1-protonated Hpyr^+^ cation, a H_2_tma^–^ monoanion and a water molecule of crystallization that is disordered over two positions. The deprotonated carboxylate group of H_2_tma^–^ forms the typical *R*_2_^2^(**8**) synthon with the N1H^+^,C2–NH_2_ site of Hpyr^+^. Pairwise hydrogen bonding between one of the two protonated carboxyl groups and the N3,C4–NH_2_ site of another Hpyr^+^ cation gives rise to a second *R*_2_^2^(**8**) motif. In addition, three H_2_tma^–^ and one Hpyr^+^ assemble into an *R*_4_^3^(**22**) macrocycle. The disordered water molecule of crystallization accepts a hydrogen bond from a C4-amino group of Hpyr^+^.

Similar to Hpyr^+^ in the pyr salt, the Htmp^+^ cation of **2** interacts through a pair of N1H^+^,C2–NH_2_···^–^OOCR hydrogen bonds with the anionic carboxylate group of H_2_tma^–^ and through a pair of N3···HO–C/C4–NH_2_···O=C hydrogen bonds with one of the protonated carboxyl groups. The other protonated carboxyl group forms a hydrogen bond with one of the methoxy groups of a neighboring Htmp^+^. The water molecules one of which is disordered over two positions are involved in hydrogen bonding with the amino group at C4, methoxy oxygen and with each other.

The two Hpyr^+^ cations (denoted A and B) of the 2:1 salt **3** are connected to the dianion of the tricarboxylic acid through the usual pair of C2–NH_2_,N1H^+^···^–^OOC hydrogen bonds. The amino groups of Hpyr^+^ A form a pair of hydrogen bonds with one oxygen of a COO^–^ group and with the carbonyl oxygen of COOH of an adjacent Htma^2–^ which generates a *R*_2_^2^(**14**) motif. The COOH group also donates a hydrogen bond to a water molecule of crystallization. Two Hpyr^+^ A···Htma^2–^ dimers assemble into a quadruple hydrogen bonding pattern in an array of *R*_2_^2^(**8**)–*R*_4_^2^(**8**)–*R*_2_^2^(**8**) rings. The C2–NH_2_···N3 *R*_2_^2^(**8**) homosynthon is observed between neighboring Hpyr^+^ B cations. The hydrogen bonding pattern in the analogous 2:1 Htmp^+^ salt **5** is different except for the common interaction of the carboxylate group with the N1H^+^,C2–NH_2_ site. The two Htmp^+^ cations interact with each other through pairwise C2–NH_2_···N3/N3···H_2_N–C4 hydrogen bonding. COOH···^–^OOC hydrogen bonds between adjacent Htma^2–^ anions give rise to a *R*_2_^2^(**16**) motif. Further hydrogen bonding occurs between water of crystallization and COO^–^, methoxy oxygen and the amino group at C4.

In the 3:1 salt **5** the tricarboxylate anion is surrounded by three Hpyr^+^ cations with pairs of hydrogen bonds between COO^–^ and the protonated N1H^+^,C2–NH_2_ sites. Furthermore, the *R*_2_^2^(**8**) synthon between the N3,C4–NH_2_ and N3,C2–NH_2_ sites of two adjacent Hpyr^+^ cations is present. The methanol molecule participates in hydrogen bonding with a carboxylate group of tma^3–^ and the amino group at C4 of Hpyr^+^. The acetonitrile molecule accepts a hydrogen bond from an amino group of one of the Hpyr^+^ cations. Hydrogen bonding between CH_3_OH, tma^3–^ and Hpyr^+^ and between CH_3_OH and two Hpyr^+^ generates a *R*_3_^3^(**16**) and *R*_3_^2^(**8**) motif, respectively.

### Ternary Salt Cocrystals of H_3_tma with Pyr or Tmp and a Pyridine Coformer

The structures of the binary 1:1 and 2:1 salts have carboxyl groups that do not participate in strong, charge-assisted hydrogen bonding with the N1H^+^,C2–NH_2_ site of the 2,4-diaminopyrimidine, but interact with the methoxy oxygen of tmp, carbonyl oxygen of another carboxylic acid molecule or oxygen of water of crystallization. We were curious if we could replace the COOH···OCH_3_, COOH···O=C and COOH···OH_2_ hydrogen bonds with the COOH···N_pyridine_ synthon and thus construct three-component crystals.

We used bipy, ebipy, pbipy, and phpy as pyridine coformers (Figure 1c). Solution crystallization experiments with mixtures containing H_3_tma, a pyridine coformer, tmp and pyr gave three new ternary salt cocrystals; (Hpyr^+^)_2_Htma^2–^·ebipy·H_2_O·CH_3_OH (**6**), (Htmp^+^)_2_Htma^2–^·1.5bipy·4H_2_O (**7**), and (Htmp^+^)_3_tma^3–^·pbipy·7H_2_O (**8**). Three more ternary salt cocrystals, (Hpyr^+^)_2_Htma^2–^·bipy·H_2_O (**9**), Htmp^+^H_2_tma^–^·1.5bipy·H_2_O (**10**), and (Htmp^+^)_2_Htma^2–^·1.5ebipy·3H_2_O (**11**), were obtained, when 1 mol equivalent 2,4-diamino-6-hydroxypyrimidine was added to the crystallization solution. Interestingly, all six ternary salt cocrystal solvates could also be prepared by liquid-assisted milling of physical mixtures of the components in the respective stoichiometric ratio and in the presence of traces of methanol and/or water. A comparison of the XRPD patterns of the milled samples with the theoretical patterns calculated from the single crystal structures is shown in the Supporting Information (Figures S8–S13). A few reports and studies on the preparation of (solvent-free) three-component cocrystals by neat- or liquid-assisted grinding can be found in the literature.^17,75−78^ However, mechanochemical ternary cocrystal formation is significantly less frequently reported than comilling of two components.

**Figure 3:**
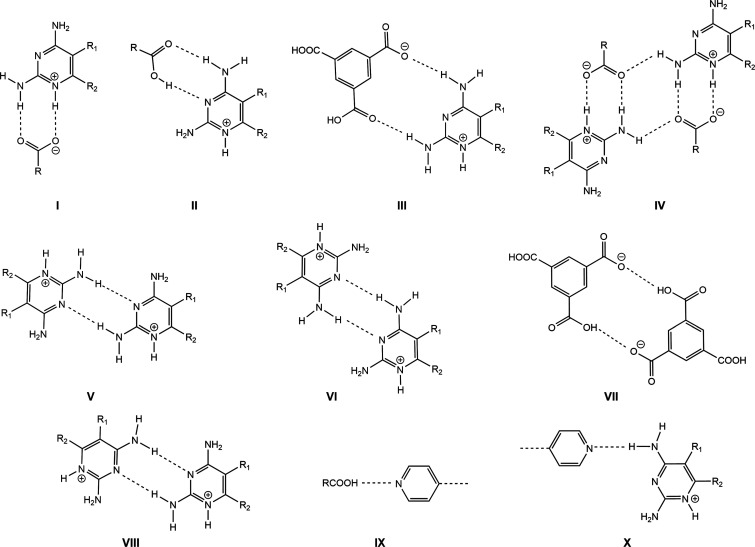
Main synthons observed in the ternary and quaternary salts and salt cocrystals.

In all structures, the Htmp^+^ or Hpyr^+^ cation interacts with the mono-, di- or trianion of H_3_tma through the expected C2–NH_2_,N1H^+^···^–^OOC *R*_2_^2^(8) motif. The main synthons observed in all the salts and salt cocrystals are listed in Table 1 and Figure 3.

**Table 1:** Main Synthons Observed in the Binary, Ternary and Quaternary Salts and Salt Cocrystals

salt/salt cocrystal	synthons
Hpyr^+^H_2_tma^–^·H_2_O (**1**)	I, II
Htmp^+^H_2_tma^–^·3H_2_O (**2**)	I, II
(Hpyr^+^)_2_Htma^2–^·3H_2_O (**3**)	I, III, IV, V
(Hpyr^+^)_3_tma^3–^·CH_3_CN·CH_3_OH·2.5H_2_O (**4**)	I, IX
(Htmp^+^)_2_Htma^2–^·5H_2_O (**5**)	I, IX, X
(Hpyr^+^)_2_tHtma^2–^·ebipy·H_2_O·CH_3_OH (**6**)	I, III, IV, VII, VIII
(Htmp^+^)_2_Htma^2–^·1.5bipy·4H_2_O (**7**)	I, III, IV, VII, VIII
(Htmp^+^)_3_tma^3–^·pbipy·7H_2_O (**8**)	I, V, IX
(Hpyr^+^)_2_Htma^2–^·bipy·H_2_O (**9**)	I, III, IV, VII, VIII
Htmp^+^H_2_tma^–^·1.5bipy·H_2_O (**10**)	I, III, IV, VII
(Htmp^+^)_2_Htma^2–^·1.5ebipy·3H_2_O (**11**)	I, III, IV, VII, VIII
Hpyr^+^Htmp^+^Htma^2–^ (**12**)	I, X
(Hpyr^+^)_2_Htmp^+^tma^3–^·2CH_3_ OH·2H_2_O (**13**)	I, V, IX
Hpyr^+^H_2_tma^–^·phpy·pbipy (**14**)	I, III, IV, VII, VIII
(Htmp^+^)_2_Htma^2–^·phpy·0.5ebipy·4H_2_O (**15**)	I, III, IV, VII, VIII
Hpyr^+^Htmp^+^Htma^2–^·bipy·H_2_O (**16**)	I, III, IV, VI, VIII

**Figure 4:**
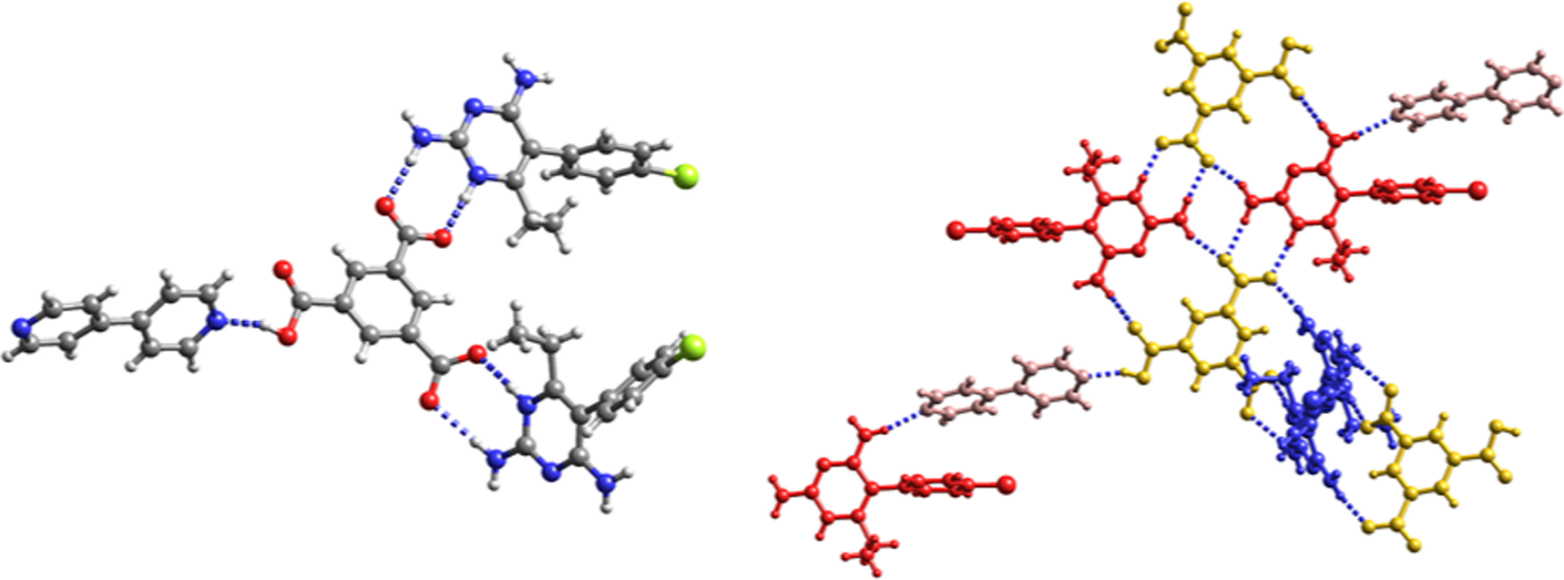
Asymmetric unit and hydrogen bonding motif of (Hpyr^+^)_2_Htma^2–^·bipy·H_2_O (**9**). The water of crystallization is not shown. Red, Hpyr^+^ A; blue Hpyr^+^ B.

As shown in Figure 4a, the Htma^2–^ dianion in **9** is surrounded by two Hpyr^+^ cations and one bipy that forms the expected COOH···N_pyridine_ synthon. Except for the common *R*_2_^2^(**8**) motif, the crystallographic environments of the Hpyr^+^ ions (denoted as A and B) are different. Hydrogen bonding between the amino protons at C2 of Hpyr^+^ A and two carboxylate oxygens of two adjacent Htma^2–^ generates the *R*_2_^2^(**8**)–*R*_4_^2^(**8**)–*R*_2_^2^(**8**)-motif (synthon IV). An additional hydrogen bond between the C4-amino group and the carbonyl oxygen of the neutral COOH group creates the *R*_2_^2^(**14**) ring motif (synthon III). Hpyr^+^ A also interacts with the second pyridine nitrogen of bipy through a C4–NH_2_···N_py_ hydrogen bond. By contrast, Hpyr^+^ B participates in only one other hydrogen bond besides the *R*_2_^2^(**8**) synthon; C4–NH_2_···^–^OOC (Figure 4b).

**Figure 5:**
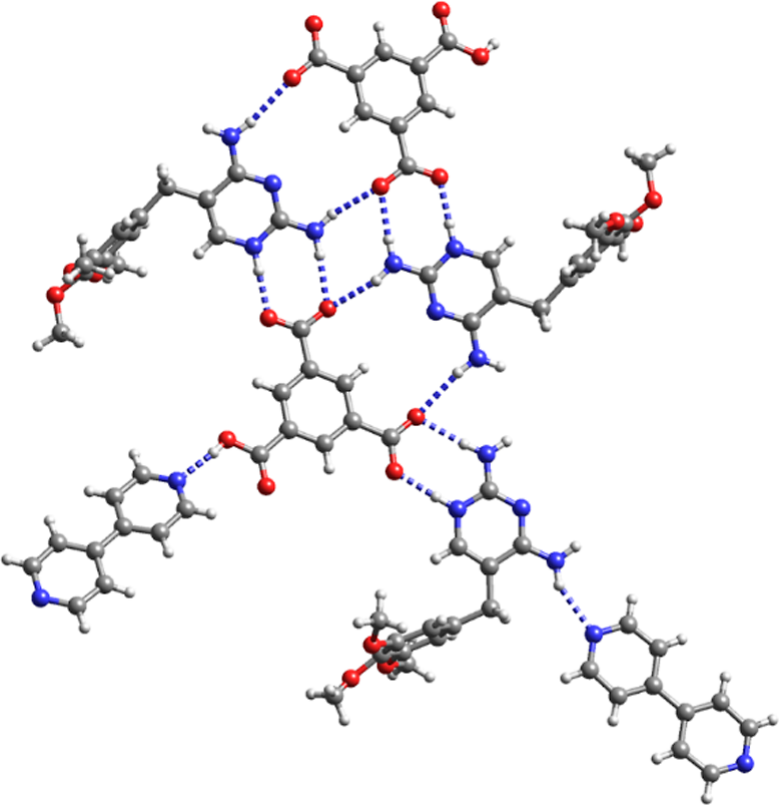
Crystal structure of (Htmp^+^)_2_Htma^2–^·1.5bipy·4H_2_O (**6**). The water molecules of crystallization are not shown.

The structure of **6** has a similar hydrogen bonding pattern and is displayed in the Supporting Information (Figure S14). Figures 5 and S15 show views of the crystal structures of **7** and **11**. The main hydrogen bonding motifs between Htmp^+^ and Htma^2–^ are the same in both structures [*R*_2_^2^(**8**), *R*_2_^2^(**14**), *R*_4_^2^(**8**)]. In addition to the COOH···N_pyridine_ synthon, the structures contain an additional half molecule of bipy/ebipy that hydrogen bonds with the amino group at the C4 position of one of the cations.

**Figure 6:**
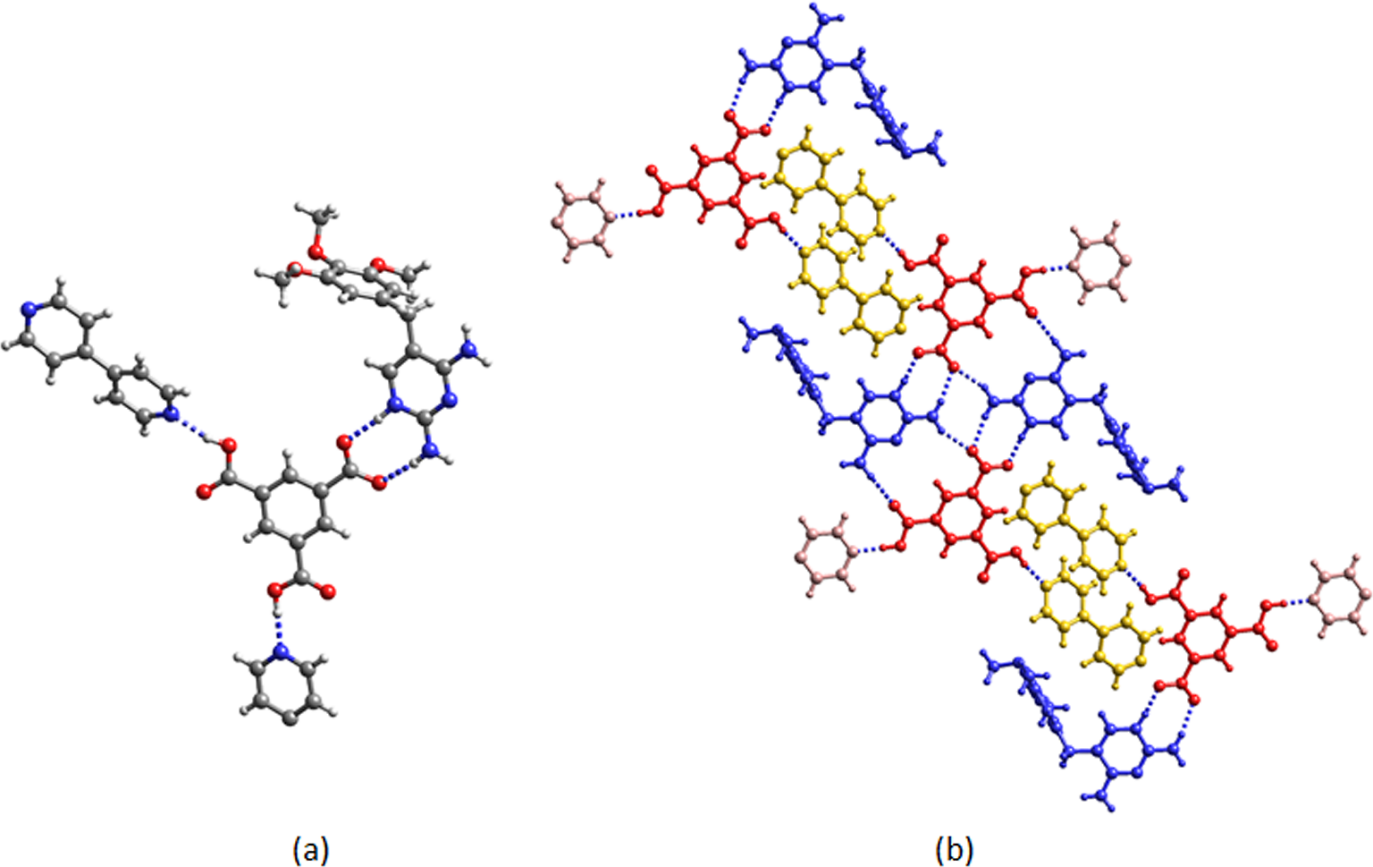
(a) Asymmetric unit and (b) hydrogen bonding motif of Htmp^+^H_2_tma^–^·1.5bipy·H_2_O (**10**). The water molecule of crystallization and the disorder of one of the bipy are not shown for clarity.

In **10** the H_2_tma^–^ monoanion is surrounded by one Htmp^+^ cation and two bipy molecules one of which lies on an inversion center (Figure 6a). Besides the COOH···N_pyridine_ synthon, synthons III and IV are observed. The water molecule of crystallization interacts with carboxylate oxygen, pyridine nitrogen and the C4-amino group of Htmp^+^ (Figure 6b).

**Figure 7:**
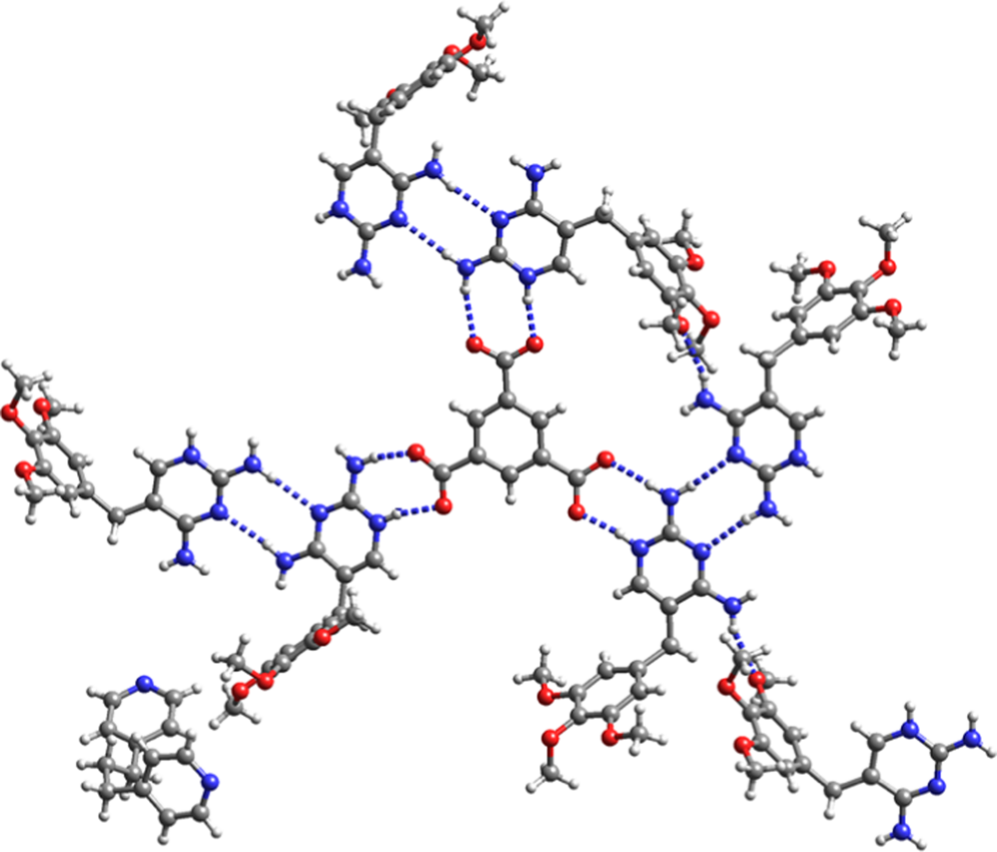
Crystal structure of (Htmp^+^)_3_tma^3–^·pbipy·7H_2_O (**8**). The water molecules of crystallization and the disorder of the methoxy groups are not shown.

In **8**, the three carboxylate groups of tma^3–^ interact with the C2–NH_2_,N1H^+^ sites of three Htmp^+^ cations (denoted A, B, and C, Figure 7). Different Htmp^+^*R*_2_^2^(**8**) homosynthons are observed. Two Htmp^+^ A cations interact with each other through their N3,C2–NH_2_ sites. Htmp^+^ B and Htmp^+^ C form pairs of C2–NH_2_···N3 and C4–NH_2_···N3 hydrogen bonds. The C4-amino group of Htmp^+^ A donates a hydrogen bond to one of the methoxy groups of Htmp^+^ B. In contrast to all other structures, the pyridine coformer does not participate in hydrogen bonding, but seems to just fill void space. The water molecules of crystallization interact with each other and with amino protons.

### Ternary Salts of H_3_tma with Pyr and Tmp

**Figure 8:**
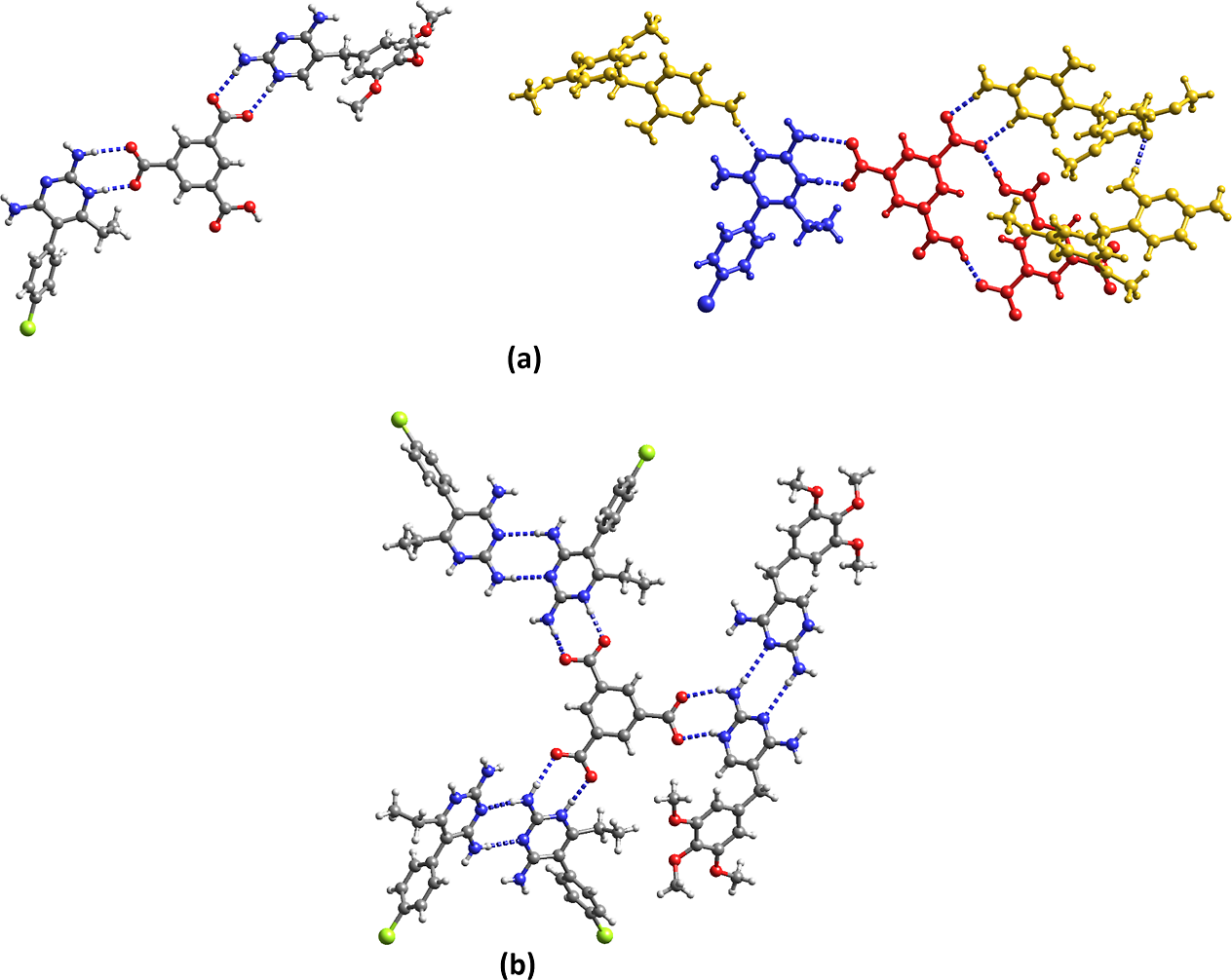
(a) Asymmetric unit and hydrogen bonding pattern of Hpyr^+^Htmp^+^Htma^2–^ (**12**). Red: Htma^2–^; blue: Hpyr^+^; yellow Htmp^+^. (b) Hydrogen bonding pattern of (Hpyr^+^)_2_Htmp^+^tma^3–^·2CH_3_OH·2H_2_O (**13**). For clarity, solvent molecules are not shown in (b).

In the next step of our study we explored the possibility of isolating three-component cocrystals or salts that contain both 2,4-diaminopyrimidines. The C5- and C6-positions in pyr and tmp have quite different substituents with distinct space and packing requirements. Furthermore, tmp has additional hydrogen bonding functionality, as the methoxy groups can act as hydrogen bond acceptors. Hence, pyr and tmp are not suitable candidates for shape-size/packing mimicry and the presence of various (self-complementary) hydrogen bond donor and acceptor sites is not favorable for selective higher order cocrystal/salt formation. Notwithstanding, we were able to crystallize the ternary salts Hpyr^+^Htmp^+^Htma^2–^ (**12**) and (Hpyr^+^)_2_Htmp^+^tma^3–^·2CH_3_OH·2H_2_O (**13**) from 1:1:1 and 2:1:1 mixtures (pyr/tmp/H_3_tma molar ratio) in methanol (Figure 8a,b). Liquid-assisted grinding gave samples that showed an amorphous halo in the XRPD analysis (Figures S16 and S17). A comparison of the XRPD pattern of the crystalline sample isolated from solution with the theoretical pattern calculated from the single crystal data confirmed that the structure of **12** was representative of the bulk sample (Figure S18). In the case of **13**, the XRPD pattern of the bulk sample (Figure S19) showed additional peaks that could not be identified.

In **12** the dianionic Htma^2–^ acts as a bridge between the two distinct cations. Two Htma^2–^ dianions form a *R*_2_^2^(16) ring by interacting with each other through COOH···^–^OOC hydrogen bonds. There is also hydrogen bonding between one of the methoxy groups and the amino group at C4 of two adjacent Htmp^+^ and between the C2-amino group of Htmp^+^ and N3 of Hpyr^+^. The crystal structure of **13** shows a tricarboxylate anion surrounded by two Hpyr^+^ homodimers and one Htmp^+^ homodimer. The Htmp^+^ homodimer is held together through a pair of C2–NH_2_···N3 hydrogen bonds, whereas the Hpyr^+^ cations interact with each other through a pair of C2–NH_2_···N3,C4–NH_2_···N3 hydrogen bonds. The water molecules of crystallization form hydrogen bonds with the C4-amino group of Hpyr^+^, while the methanol molecules interact with carboxylate oxygen and the amino group at C2 of Htmp^+^.

### Quaternary Salt Cocrystals of H_3_tma, Tmp, Pyr and Pyridine Coformers

**Figure 9:**
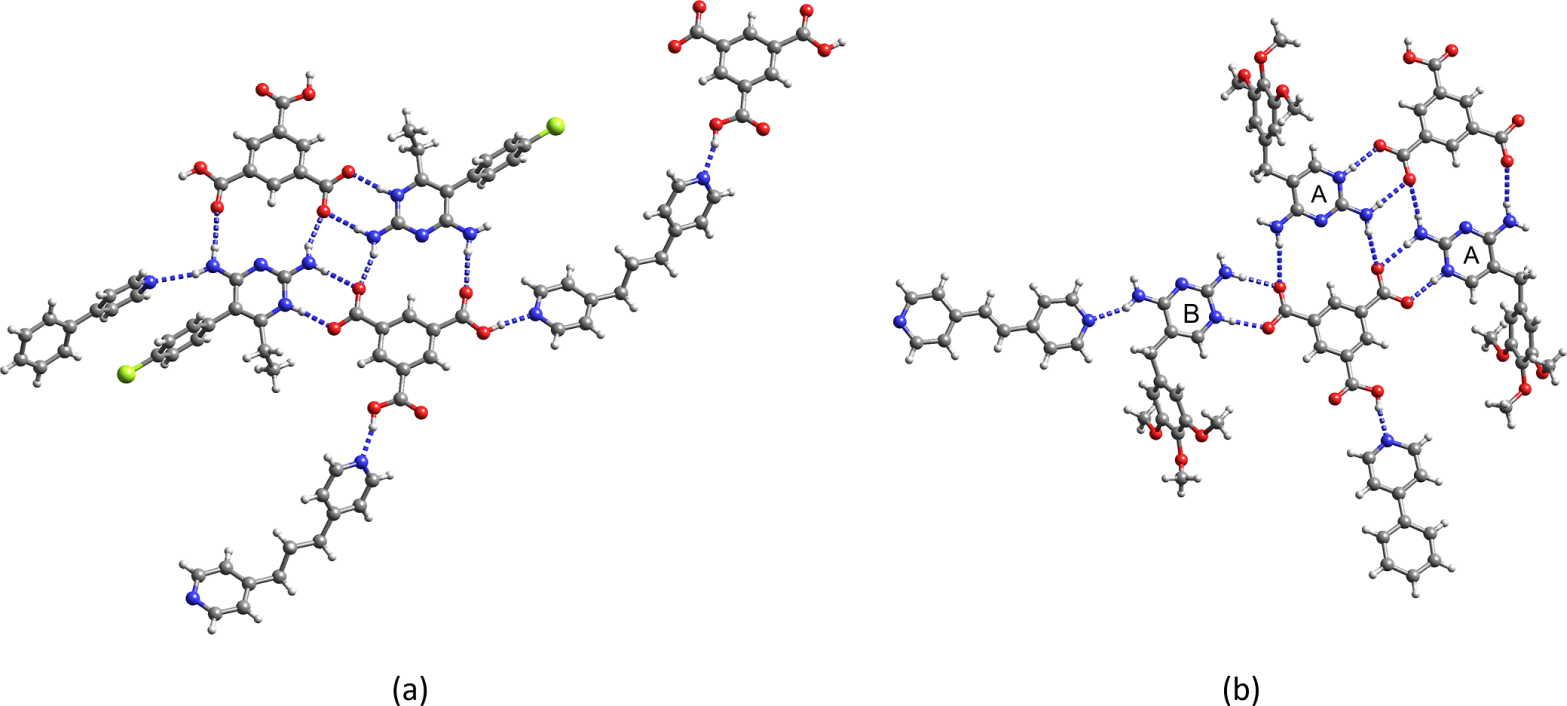
Crystal structure of (a) Hpyr^+^H_2_tma^–^·phpy·pbipy (**14**) and (b) (Htmp^+^)_2_Htma^2–^·phpy·0.5ebipy·4H_2_O (**15**). The water molecules of crystallization and the disorder of the methoxy groups and of the phenyl ring of phpy are not shown for clarity.

Finally, we were able to isolate three quaternary salt cocrystals, two containing Htma^2–^, Htmp^+^ or Hpyr^+^ and two different pyridine coformers and one containing Htma^2–^, bipy and both 2,4-diaminopyrimidines. The hydrogen bonding in Hpyr^+^H_2_tma^–^·phpy·pbipy (**14**) and (Htmp^+^)_2_Htma^2–^·phpy·0.5ebipy·4H_2_O (**15**) is displayed in Figure 9a,b, respectively. In the former, the H_2_tma^–^ monoanion interacts with Hpyr^+^ through synthons I [*R*_2_^2^(**8**)] and III [*R*_2_^2^(**14**)] and with pbipy. Both nitrogens of pbipy form COOH···N_pyridine_ hydrogen bonds, bridging two H_2_tma^–^·Phpy binds to Hpyr^+^ via a C4–NH_2_···N_pyridine_ hydrogen bond. Furthermore, the quadruple hydrogen bonding array between two cations and two anions (synthon IV) is observed.

The two cations of **15** (denoted Htmp^+^ A and B) have distinct crystallographic environments. Htmp^+^ A interacts with two Htma^2–^ through C2–NH_2_,N1H^+^···^–^OOC [*R*_2_^2^(**8**)] and C2–NH_2_,C4–NH_2_···^–^OOC,^–^OOC [*R*_2_^2^(**14**)] hydrogen bond pairs. The *R*_4_^2^(**8**) motif between two Htmp^+^ A and two Htma^2–^ is also observed. Htmp^+^ B interacts with Htma^2–^ (C2–NH_2_,N1H^+^···^–^OOC) and with ebipy (C4–NH_2_···N_pyridine_). The pyridine nitrogen of phpy forms a hydrogen bond with the carboxyl group of Htma^2–^.

**Figure 10:**
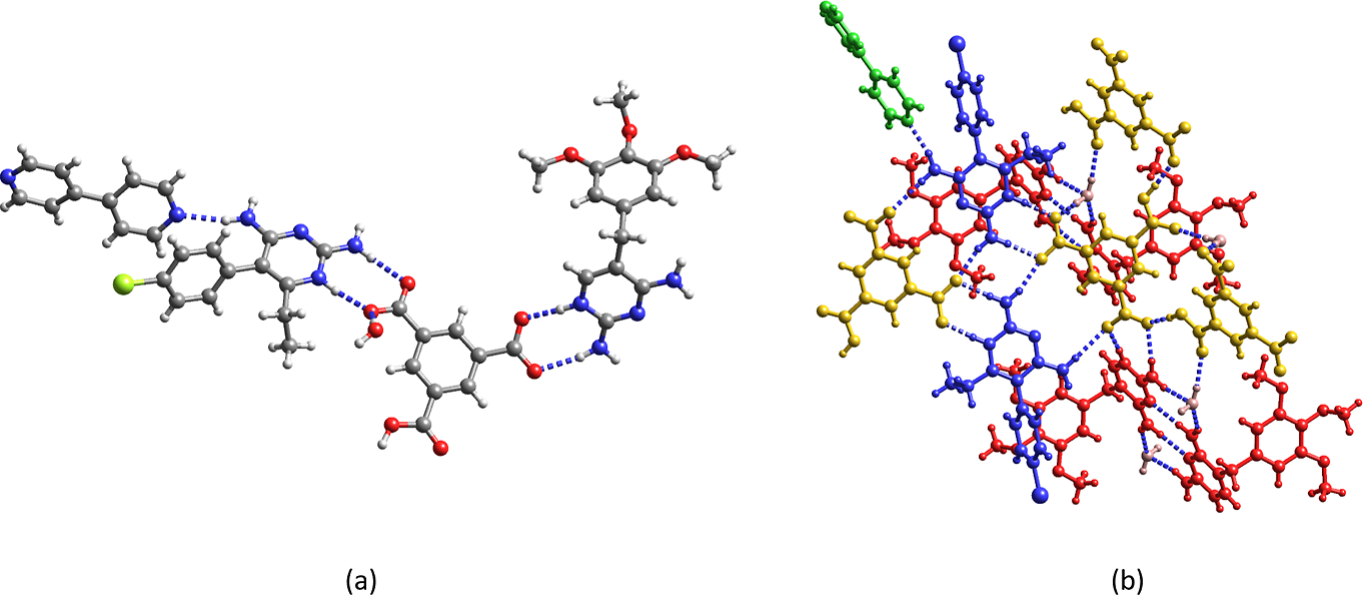
(a) Asymmetric unit and (b) hydrogen bonding pattern of Hpyr^+^Htmp^+^Htma^2–^·bipy·H_2_O (**16**). Red: Htmp^+^; blue: Hpyr^+^; yellow Htma^2–^; green bipy.

The asymmetric unit of the third quaternary salt cocrystal **16** consists of a Htmp^+^ cation, a Hpyr^+^ cation, a Htma^2–^ dianion, a bipy and a water molecule of crystallization (Figure 10a). The anionic carboxylate groups of Htma^2–^ interact with Htmp^+^ and Hpyr^+^ while bipy interacts with the C4-amino group of Hpyr^+^ (Figure 10). Hpyr^+^ also forms the *R*_2_^2^(**14**) motif with Htma^2–^ and the *R*_4_^2^(**8**) motif is observed between two Hpyr^+^ and two Htma^2–^. Water and two Htma^2–^ anions assemble into an 18-membered ring [*R*_3_^3^(**18**)]. The Htmp^+^ C4–NH_2_···N3 homosynthon is present and is stabilized by two “bridging” water molecules that hydrogen bond with the amino groups at C2 and C4. Hydrogen bonding between water, Htmp^+^ and the COOH group of Htma^2–^ generates an *R*_4_^3^(**10**) motif (Figure 10b).

**Figure 11:**
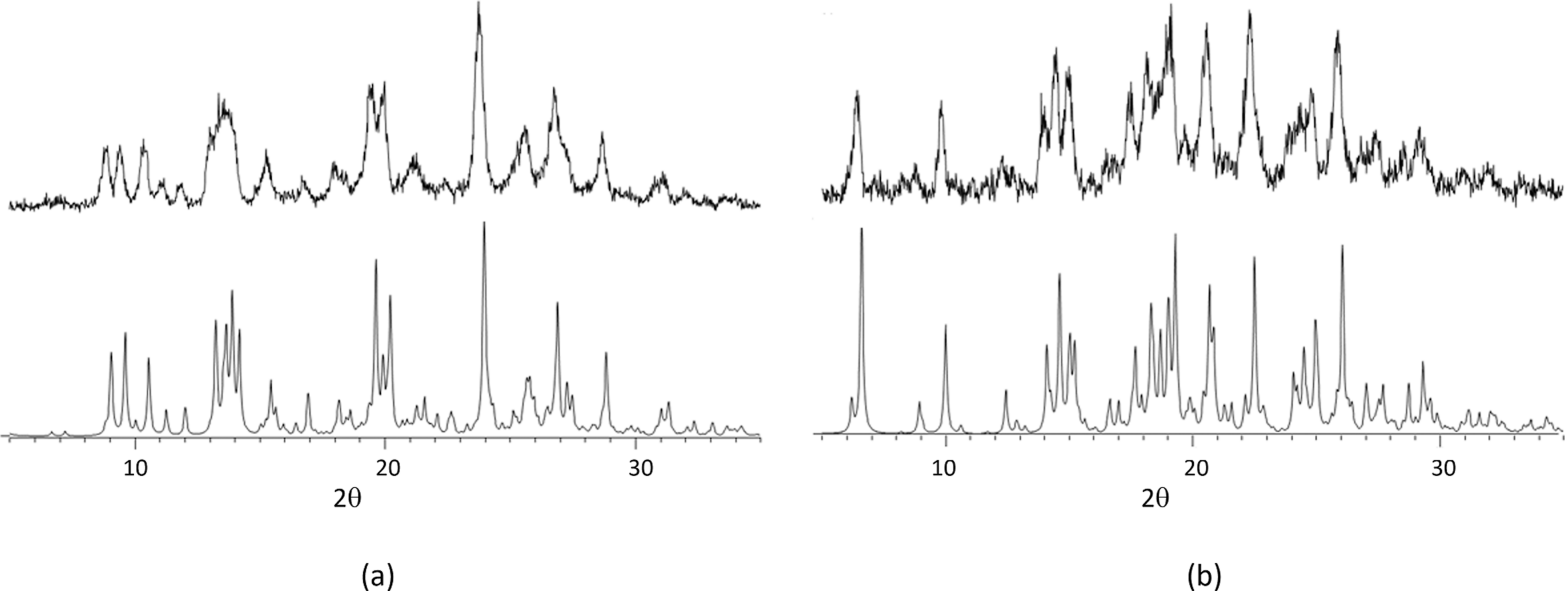
(a) XRPD pattern of a 2:1:1:0.5 mixture of tmp, H_3_tma, phpy and ebipy after milling for 30 min in the presence of traces of methanol and water (top) and the theoretical XRPD pattern of (Htmp^+^)_2_Htma^2–^·phpy·0.5ebipy·4H_2_O calculated from the single crystal data. (b) XRPD pattern of a 1:1:1:1 mixture of pyr, tmp, H_3_tma and bipy after milling for 30 min in the presence of traces of methanol and water (top) and the theoretical XRPD pattern of Hpyr^+^Htmp^+^Htma^2–^·bipy·H_2_O calculated from the single crystal data (bottom).

Remarkably, all three quaternary salt cocrystals could be obtained by liquid-assisted grinding of the respective stoichiometric mixtures of the components (Figures 11 and S20). In all grinding experiments (including the ones to prepare binary and ternary systems) traces of the solvent or the solvent mixture that yielded single crystals during solution crystallization were added. It is noteworthy that the binary, ternary and quaternary salts all bar two crystallized as hydrates or mixed hydrate/solvates. After formation of all coformer interactions, there is still unused hydrogen bonding capacity left which may promote the inclusion of water and solvent molecules. Furthermore, the “unsymmetric” decoration of H_3_tma with two or three different coformers may lead to an inefficient packing with voids that are filled with water/solvent to stabilize the structure. The mixtures containing the four components in the correct stoichiometric ratios convert to the phase-pure quaternary salt cocrystals on milling, as this generates the maximum number of strong NH^+^,NH_2_···^–^OOC, NH_2_···N_pyridine_ and COOH···N_pyridine_ hydrogen bonds. Furthermore, the mechanochemical synthesis of four-component cocrystals is not affected by the relative solubilities of potential lower-order cocrystals. The fact that pure quaternary multicomponent salts could be achieved highlights the importance and potential of liquid-assisted grinding as a solid-state preparation technique.

### Thermal Analysis

The milled samples were further analyzed by DSC and the plots are shown in Figures S21–S35. For the anhydrous cocrystal **14** a single thermal event is observed at 180.3 °C corresponding to melting. The DSC plot of the amorphous solid that resulted from ball-milling the components of **12** in a stoichiometric ratio shows a recrystallization exotherm at 165.2 °C and a melting endotherm at 253.2 °C. Likewise, the amorphous samples obtained after milling physical mixtures of the components of **2** and **5** featured a recrystallization exotherm (176.8 °C (**2**) and 157.9 °C (**5**)) and a single endotherm assigned to melting of the salts at 285.1 °C (**2**) and 257.3 °C (**5**). When a 2:1:1 mixture of pyr, tmp and H_3_tma was milled in the presence of traces of methanol and water (the composition of **13**), an exotherm at 148.8 °C and two endotherms at 194.3 and 245.8 °C were observed, indicating that the amorphous sample does not crystallize to the phase-pure ternary cocrystal on heating. The DSC plot of **4** prepared by milling featured three broad endotherms and an exotherm in the 50–170 °C region that are assigned to solvent loss followed by phase transformation. The loss of solvent was confirmed by thermogravimetric analysis. Furthermore, two endothermic peaks at 240.9 and 285.5 °C were observed. The peak at 240.9 °C that can be assigned to the melting of pyr indicates that solvent loss does not give the dehydrated salt. Likewise, DSC and thermogravimetric analysis of **8** show the loss of water and the collapse of the ternary cocrystal structure. **6** is also not stable on heating. Solvent loss gives rise to a very broad endotherm around 120 °C. The next two endothermic peaks appear at the melting temperature of ebipy and pyr, respectively. An endotherm at 263.5 °C is assigned to the melting of the new phase formed on loss of the solvent, ebipy and pyr. Likewise, the DSC analyses of **7**, **9**, **10**, **11**, **15**, and **16** show several endothermic events after solvent loss indicating that desolvation destabilizes the lattices of the ternary and quaternary salt cocrystals. The DSC plot of **1** is consistent with loss of water around 50–110 °C followed by phase transformation at 163.5 °C and melting of the desolvated salt at 310.3 °C.

## Conclusions

Trimesic acid readily forms salt structures in which its mono-, di- or trianion is “surrounded” by different diaminopyrimidine and pyridyl coformers, leading to a large variety of three- and four-component cocrystals. The facile formation of ternary and quaternary salt cocrystals with different combinations of diaminopyrimidines and/or pyridyls can be rationalized by (1) the observation that proton transfer between H_3_tma and the diaminopyrimidines results in binary systems that are soluble in polar solvents and (2) the fact that the pyridyl coformers have only hydrogen bonding acceptor functionalities. As the pyridyls cannot form hydrogen bonds with each other, the crystallization of a higher-order salt cocrystal from a stoichiometric mixture is the only possibility to maximize hydrogen bonding and is therefore favored over the crystallization of a mix of binary salts or cocrystals. This also allows for the mechanochemical synthesis of all three- and four-component crystals described in this work.

## Supporting Information Available

Additional figures of X-ray structures, XRPD patterns, details on the solution crystallization and milling experiments (PDF)
