# Supplementary material for: Trimesic Acid as a Building Block for Ternary and Quaternary Salts and Salt Cocrystals
Source: Cryst Growth Des. 2024 Oct 31;24(22):9403–14. doi: 10.1021/acs.cgd.4c00779 (PMC11583203; doi:10.1021/acs.cgd.4c00779)
Supplement: Supplementary file 1 — cg4c00779_si_001.pdf [file cg4c00779_si_001.pdf]

Trimesic Acid as a Building Block for Ternary and Quaternary Salts and Salt Cocrystals

Lamis Alaa Eldin Refat,[1,2] Marwah Aljohani[3] and Andrea Erxleben[1,2,*]

[1] School of Biological and Chemical Sciences, University of Galway, Galway, H91TK33, Ireland

[2] Synthesis and Solid State Pharmaceutical Centre (SSPC), Limerick, V94 T9PX, Ireland

[3] Department of Chemistry, College of Science, Imam Abdulrahman Bin Faisal University, P.O. Box 76971, Dammam 31441, Saudi Arabia

*Corresponding author email address: andrea.erxleben@nuigalway.ie (AE)

Supporting Information

Table S1. Solution crystallization experiments

Component 1	Component 2	Component 3	Component 4	Component 5	Molar ratio	Solvent	Result
25.0 mg pyr	21.0 mg H_3tma	--	--	--	1:1	C_2H_5OH	$Hpyr^+H_2tma^-\cdot H_2O$
25.0 mg tmp	18.1 mg H_3tma	--	--	--	1:1	CH_3OH	$Htmp^+H_2tma^-\cdot 3H_2O$
25.0 mg pyr	10.5 mg H_3tma	--	--	--	2:1	CH_3OH	$(Hpyr^+)_2Htma^{2-}\cdot 3H_2O$
25.0 mg tmp	9.1 mg H_3tma	--	--	--	2:1	CH_3OH	Very thin non-diffracting needles
37.5 mg pyr	10.5 mg H_3tma	--	--	--	3:1	CH_3OH + CH_3CN	$(Hpyr^+)_3tma^{3-}$ $\cdot CH_3CN\cdot CH_3OH\cdot 2.5H_2O$
25.0 mg tmp	6.0 mg H_3tma	--	--	--	3:1	CH_3OH	$(Htmp^+)_2Htma^{2-}\cdot 5H_2O$
20.0 mg pyr	16.9 mg H_3tma	23.3 mg tmp	--	--	1:1:1	CH_3OH	$Hpyr^+Htmp^+Htma^{2-}$
34.2 mg pyr	14.5 mg H_3tma	20.0 mg tmp	--	--	2:1:1	CH_3OH	$(Hpyr^+)_2Htmp^+tma^{3-}\cdot 2CH_3OH\cdot 2H_2O$
17.1 mg pyr	14.5 mg H_3tma	40.0 mg tmp	--	--	1:1:2	CH_3OH	$(Hpyr^+)_2Htmp^+tma^{3-}\cdot 2CH_3OH\cdot 2H_2O$
20.0 mg tmp	14.5 mg H_3tma	17.1 mg pyr	12.5 mg ebipy	--	1:1:1:1	CH_3OH	$(Hpyr^+)_2Htma^{2-}\cdot$ebipy$\cdot H_2O\cdot CH_3OH$
20.0 mg tmp	14.5 mg H_3tma	17.1 mg pyr	10.7 mg bipy	--	1:1:1:1	CH_3OH	$(Htmp^+)_2Htma^{2-}\cdot$1.5bipy$\cdot 4H_2O$
20.0 mg tmp	14.5 mg H_3tma	10.7 mg bipy	8.7 mg 2,4dahp*	--	1:1:1:1	DMF	$Htmp^+H_2tma^-\cdot$1.5bipy$\cdot H_2O$
20.0 mg tmp	14.5 mg H_3tma	17.1 mg pyr	13.6 mg pbipy	--	1:1:1:1	CH_3OH	$(Htmp^+)_3tma^{3-}\cdot$pbipy$\cdot 7H_2O$
20.0 mg tmp	14.5 mg H_3tma	17.1 mg pyr	21.4 mg bipy	8.7 mg 2,4dahp*	1:1:1:2:1	CH_3OH	$(Hpyr^+)_2Htma^{2-}\cdot$bipy$\cdot H_2O$
20.0 mg tmp	14.5 mg H_3tma	17.1 mg pyr	12.5 mg ebipy	8.7 mg 2,4dahp*	1:1:1:1:1	CH_3OH + CH_3CN	$(Htmp^+)_2Htma^{2-}\cdot$1.5ebipy$\cdot 3H_2O$
20.0 mg tmp	14.5 mg H_3tma	17.1 mg pyr	12.5 mg ebipy	10.7 mg phpy	1:1:1:1:1	CH_3OH + CH_3CN	$(Htmp^+)_2Htma^{2-}$ $\cdot$phpy$\cdot$0.5ebipy$\cdot 4H_2O$
20.0 mg tmp	14.5 mg H_3tma	17.1 mg pyr	10.7 mg bipy	10.7 mg phpy	1:1:1:1:1	CH_3OH	$Hpyr^+Htmp^+Htma^{2-}\cdot$bipy$\cdot H_2O$
20.0 mg tmp	14.5 mg H_3tma	17.1 mg pyr	13.6 mg pbipy	10.7 mg phpy	1:1:1:1:1	CH_3OH	$Hpyr^+H_2tma^-\cdot$phpy$\cdot$pbipy

* 2,4-diamino-6-hydroxypyrimidine

Table S2. Sample compositions in the ball-milling experiments.

Cocrystal	Component 1	Component 2	Component 3	Component 4	Molar ratio	Solvent
$Hpyr^+H_2tma^-\cdot H_2O$	150 mg pyr	126.65 mg H_3tma	--	--	1:1	50 µL C_2H_5OH
$Htmp^+H_2tma^-\cdot 3H_2O$	200 mg tmp	145 mg H_3tma	--	--	1:1	25 µL CH_3OH + 25 µL H_2O
$(Hpyr^+)_2Htma^{2-}\cdot 3H_2O$	150 mg pyr	63.32 mg H_3tma	--	--	2:1	25 µL CH_3OH + 25 µL H_2O
$(Htmp^+)_2Htma^{2-}\cdot 5H_2O$	200 mg tmp	72.5 mg H_3tma	--	--	2:1	25 µL CH_3OH + 25 µL H_2O
$(Hpyr^+)_3tma^{3-}\cdot CH_3CN\cdot CH_3OH\cdot 2.5H_2O$	200 mg pyr	56.3 mg H_3tma	--	--	3:1	15 µL CH_3OH + 15 µL CH_3CN + 15 µL H_2O
$Hpyr^+Htmp^+Htma^{2-}$	150 mg tmp	108.5 mg H_3tma	128.5 mg pyr	--	1:1:1	50 µL CH_3OH
$(Hpyr^+)_2Htmp^+tma^{3-}\cdot 2CH_3OH\cdot 2H_2O$	150 mg tmp	108.5 mg H_3tma	257 mg pyr	--	1:1:2	25 µL CH_3OH + 25 µL H_2O
$(Hpyr^+)_2Htma^{2-}\cdot$ebipy$\cdot H_2O\cdot CH_3OH$	200 mg pyr	84.5 mg H_3tma	73.3 mg ebipy	--	2:1:1	25 µL CH_3OH + 25 µL H_2O
$(Htmp^+)_2Htma^{2-}\cdot$1.5bipy$\cdot 4H_2O$	200 mg tmp	72.3 mg H_3tma	161.39 mg bipy	--	2:1:1.5	50 µL H_2O
$Htmp^+H_2tma^-\cdot$1.5bipy$\cdot H_2O$	150 mg tmp	108.5 mg H_3tma	121 mg bipy	--	1:1:1.5	25 µL CH_3OH + 25 µL H_2O
$(Htmp^+)_3tma^{3-}\cdot$pbipy$\cdot 7H_2O$	200 mg tmp	48.2 mg H_3tma	45.5 mg pbipy	--	3:1:1	50 µL H_2O
$(Hpyr^+)_2Htma^{2-}\cdot$bipy$\cdot H_2O$	200 mg pyr	84.5 mg H_3tma	63 mg bipy	--	2:1:1	25 µL CH_3OH + 25 µL H_2O
$(Htmp^+)_2Htma^{2-}\cdot$1.5ebipy$\cdot 3H_2O$	200 mg tmp	72.3 mg H_3tma	141.5 mg ebipy	--	2:1:1.5	25 µL CH_3OH + 25 µL H_2O
$(Htmp^+)_2Htma^{2-}\cdot$phpy$\cdot$0.5ebipy$\cdot 4H_2O$	200 mg tmp	72.3 mg H_3tma	53.5 mg phpy	47 mg ebipy	2:1:1:0.5	25 µL CH_3OH + 25 µL H_2O
$Hpyr^+Htmp^+Htma^{2-}\cdot$bipy$\cdot H_2O$	150 mg tmp	108.5 mg H_3tma	128.5 mg pyr	80.7 mg bipy	1:1:1:1	25 µL CH_3OH + 25 µL H_2O
$Hpyr^+H_2tma^-\cdot$phpy$\cdot$pbipy	150 mg pyr	126.65 mg H_3tma	119.5 mg pbipy	93.6 mg phpy	1:1:1:1	25 µL CH_3OH + 25 µL H_2O

Table S3. Crystal data of $Hpyr^+H_2tma^-\cdot H_2O$, $(Hpyr^+)_2Htma^{2-}\cdot 3H_2O$, $(Hpyr^+)_3tma^{3-}\cdot CH_3CN\cdot CH_3OH\cdot 2.5H_2O$, $Htmp^+H_2tma^-\cdot 3H_2O$, and $(Htmp^+)_2Hma^{2-}\cdot 5H_2O$.

	$Hpyr^+H_2tma^-\cdot H_2O$	$(Hpyr^+)_2Htma^{2-}\cdot 3H_2O$	$(Hpyr^+)_3tma^{3-}\cdot CH_3CN$ $\cdot CH_3OH\cdot 2.5H_2O$	$Htmp^+H_2tma^-\cdot 3H_2O$	$(Htmp^+)_2Hma^{2-}\cdot 5H_2O$
Formula	$C_{21}H_{21}ClN_4O_7$	$C_{33}H_{38}Cl_2N_8O_9$	$C_{48}H_{57}Cl_3N_{13}O_{9.5}$	$C_{23}H_{30}N_4O_{12}$	$C_{37}H_{52}N_8O_{17}$
M_r	476.87	761.612	1074.41	554.51	880.87
Crystal color and habit	Colorless block	Colorless block	Colorless plate	Colorless plate	Colorless block
Crystal size (mm)	0.5 x 0.2 x 0.15	0.6 x 0.3 x 0.3	0.8 x 0.8 x 0.2	0.4 x 0.2 x 0.05	0.5 x 0.2 x 0.1
Crystal system	Monoclinic	Monoclinic	Triclinic	Triclinic	Triclinic
Space group	$P2_1/n$	I2/a	P-1	P-1	P-1
a [Å]	12.3470(11)	19.5185(9)	10.4833(4)	7.1003(8)	9.6020(3)
b [Å]	12.7626(5)	8.5494(5)	15.4335(6)	12.6537(17)	15.3037(6)
c [Å]	16.2531(7)	47.222(2)	17.9033(7)	15.919(2)	15.4037(6)
α [°]	90	90	78.875(3)	76.124(12)	93.523(3)
β [°]	92.451(6)	97.006(4)	77.258(3)	79.795(11)	98.505(3)
γ [°]	90	90	73.658(4)	75.012(11)	103.882(3)
V [Å^3]	2558.8(3)	7821.2(7)	2684.53(19)	1331.1(3)	2162.13(14)
Z	4	8	2	2	2
D_{calc} (g cm^{-3})	1.238	1.294	1.329	1.384	1.353
Temperature (K)	300.3(9)	297.0(1)	293.0(2)	297.0(1)	293.0(2)
No. measd. reflections	18752	33448	21107	9375	16344
No. unique refl. (R_{int})	4681	9929	9830	4691	7907
No. obs. reflections	2619	5654	6676	1881	3430
Final R_1, wR_2 (obs. refl.)	0.0974, 0.2846	0.0750, 0.2055	0.0614, 0.1562	0.0866, 0.1997	0.0752, 0.1907
Goodness-of-fit (obs. refl.)	1.104	1.027	1.022	1.006	0.943

Table S4. Crystal data of $(Hpyr^+)_2$ $Htma^{2-}$·ebipy·H_2O·CH_3OH, $Htmp^+H_2tma^-$·1.5bipy·H_2O, $(Htmp^+)_2Htma^{2-}$·1.5bipy·$4H_2O$, $(Htmp^+)_3tma^{3-}$·pbipy·$7H_2O$, $(Hpyr^+)_2Htma^{2-}$·bipy·H_2O, and $(Htmp^+)_2Htma^{2-}$·1.5ebipy·$3H_2O$.

	$(Hpyr^+)_2Htma^{2-}$ ·ebipy·H_2O·CH_3OH	$Htmp^+H_2tma^-$ ·1.5bipy·H_2O	$(Htmp^+)_2Htma^{2-}$ ·1.5bipy·$4H_2O$	$(Htmp^+)_3tma^{3-}$ ·pbipy·$7H_2O$	$(Hpyr^+)_2Htma^{2-}$ ·bipy·H_2O	$(Htmp^+)_2Htma^{2-}$ ·1.5ebipy·$3H_2O$
Formula	$C_{46}H_{48}Cl_2N_{10}O_8$	$C_{38}H_{38}N_7O_{10}$	$C_{52}H_{62}N_{11}O_{16}$	$C_{64}H_{88}N_{14}O_{22}$	$C_{43}H_{42}Cl_2N_{10}O_7$	$C_{55}H_{63}N_{11}O_{15}$
M_r	939.84	752.75	1097.12	1405.48	881.76	1118.16
Crystal color and habit	Colorless block	Colorless plate	Colorless needle	Colorless plate	Colorless plate	Pale yellow rod
Crystal size (mm)	0.6 x 0.3 x 0.2	0.6 x 0.4 x 0.05	0.6 x 0.1 x 0.1	0.6 x 0.3 x 0.1	0.4 x 0.2 x 0.05	0.7 x 0.2 x 0.2
Crystal system	Triclinic	Triclinic	Triclinic	Triclinic	Triclinic	Triclinic
Space group	P-1	P-1	P-1	P-1	P-1	P-1
a [Å]	11.2458(6)	7.9949(3)	10.7545(9)	12.8359(5)	10.4275(8)	10.5774(4)
b [Å]	11.9343(4)	10.7166(4)	14.0563(10)	13.7594(6)	11.7630(9)	14.2814(6)
c [Å]	18.0311(9)	22.3347(9)	20.5048(15)	20.8242(10)	18.1586(15)	20.6290(7)
α [°]	81.044(3)	87.939(3)	74.559(6)	78.846(4)	94.548(7)	97.473(3)
β [°]	76.804(4)	80.423(3)	79.593(7)	78.967(4)	106.387(7)	101.437(3)
γ [°]	86.679(3)	76.583(3)	68.622(7)	87.994(4)	93.615(7)	108.840(4)
V [$Å^3$]	2326.71(19)	1835.42(13)	2770.1(4)	3541.7(3)	2121.6(3)	2826.7(2)
Z	2	2	2	2	2	2
D_{calc} (g cm^{-3})	1.342	1.362	1.315	1.318	1.380	1.314
Temperature (K)	293.0(2)	293.0(2)	293.0(2)	293.0(2)	293.0(2)	293.0(2)
No. measd. reflections	17564	14164	21459	28199	16123	22135
No. unique refl. (R_{int})	8519	6708	10097	12947	7748	10336
No. obs. reflections	5681	3911	3709	4421	3182	6418
Final R_1, wR_2 (obs. refl.)	0.0617, 0.1576	0.0621, 0.1607	0.0885, 0.2002	0.0696, 0.1323	0.0779, 0.1779	0.0644, 0.1804
Goodness-of-fit (obs. refl.)	0.975	0.923	0.912	0.913	0.954	0.993

Table S5. Crystal data of $Hpyr^+Htmp^+Htma^{2-}$, $(Hpyr^+)_2Htmp^+tma^{3-}\cdot 2CH_3OH\cdot 2H_2O$, $(Htmp^+)_2Htma^{2-}\cdot phpy\cdot 0.5ebipy\cdot 4H_2O$, $Hpyr^+Htmp^+Htma^{2-}\cdot bipy\cdot H_2O$, and $Hpyr^+\cdot H_2tma^-\cdot phpy\cdot pbipy$.

	$Hpyr^+Htmp^+Htma^{2-}$	$(Hpyr^+)_2Htmp^+tma^{3-}$ $\cdot 2CH_3OH\cdot 2H_2O$	$(Htmp^+)_2Htma^{2-}$ $\cdot phpy\cdot 0.5ebipy\cdot 4H_2O$	$Hpyr^+Htmp^+Htma^{2-}$ $\cdot bipy\cdot H_2O$	$Hpyr^+\cdot H_2tma^-$ $\cdot phpy\cdot pbipy$
Formula	$C_{35}H_{37}ClN_8O_9$	$C_{49}H_{62}Cl_2N_{12}O_{13}$	$C_{54}H_{64}N_{10}O_{16}$	$C_{45}H_{47}ClN_{10}O_{10}$	$C_{45}H_{42}ClN_7O_6$
M_r	749.17	1098.00	1109.15	923.37	812.30
Crystal color and habit	Colorless diamond block	Colorless block	Colorless needle	Colorless plate	Colorless block
Crystal size (mm)	0.8 x 0.5 x 0.5	0.8 x 0.7 x 0.3	0.5 x 0.05 x 0.05	0.5 x 0.2 0.05	0.6 x 0.3 x 0.2
Crystal system	Monoclinic	Monoclinic	Triclinic	Monoclinic	Triclinic
Space group	$P2_1/c$	$P2_1/c$	P-1	$P2_1/c$	P-1
a [Å]	12.0492(4)	24.4181(9)	10.7016(9)	14.8029(9)	10.5375(5)
b [Å]	11.8949(5)	9.1803(5)	14.4384(11)	26.7680(15)	12.0159(5)
c [Å]	24.8138(11)	25.9811(10)	20.4264(15)	12.2457(4)	17.1110(7)
α [°]	90	90	97.199(6)	90	90.471(4)
β [°]	93.606(4)	102.856(4)	102.165(7)	106.105(5)	101.919(4)
γ [°]	90	90	110.206(7)	90	101.955(4)
V [Å^3]	3549.4(2)	5678.1(4)	2827.1(4)	4661.9(4)	2070.92(16)
Z	4	4	2	4	2
D_{calc} (g cm^{-3})	1.402	1.284	1.303	1.316	1.303
Temperature (K)	293.0(2)	293.0(2)	293.0(2)	293.0(2)	293.0(2)
No. measd. reflections	25173	41214	21133	35985	18789
No. unique refl. (R_{int})	6491	10356	10320	8520	9662
No. obs. reflections	5042	7273	3089	3845	5188
Final R_1, wR_2 (obs. refl.)	0.0509, 0.1240	0.0975, 0.2899	0.1076, 0.2422	0.0627, 0.1400	0.0682, 0.1584
Goodness-of-fit (obs. refl.)	1.032	1.018	0.971	0.911	1.028

Table S6. C-O bond distances (Å) of the carboxylate groups forming the $R_2^2(8)$ motif with the tmpH+ cation in the trimesic acid salts

**Compound**	**C-O bond distances** (Å)
$Hpyr^+H_2tma^-\cdot H_2O$	1.226(5)/1.232(5)
$Htmp^+H_2tma^-\cdot 3H_2O$	1.257(7)/1.254(7)
$(Hpyr^+)_2Htma^{2-}\cdot 3H_2O$	1.240(4)/1.257(3); 1.259(3)/1.241(3)
$(Htmp^+)_2Htma^{2-}\cdot 5H_2O$	1.274(4)/1.232(4); 1.267(4)/1.249(4)
$(Hpyr^+)_3tma^{3-}\cdot CH_3CN.CH_3OH\cdot 2.5H_2O$	1.255(3)/1.253(3); 1.260(3)/1.241(3); 1.254(3)/1.248(3)
$Hpyr^+Htmp^+Htma^{2-}$	1.287(3)/1.221(3); 1.275(3)/1.239(3)
$(Hpyr^+)_2Htmp^+tma^{3-}\cdot 2CH_3OH\cdot 2H_2O$	1.248(5)/1.242(5); 1.253(6)/1.258(6); 1.248(5)/1.253(5)
$(Hpyr^+)_2Htma^{2-}\cdot$ebipy$\cdot H_2O\cdot CH_3OH$	1.256(3)/1.246(3); 1.253(3)/1.237(3)
$(Htmp^+)_2Htma^{2-}\cdot$1.5bipy$\cdot 4H_2O$	1.232(5)/1.265(5); 1.249(5)/1.257(5)
$Htmp^+H_2tma^-\cdot$1.5bipy$\cdot H_2O$	1.248(3)/1.260(3)
$(Htmp^+)_3tma^{3-}\cdot$pbipy$\cdot 7H_2O$	1.258(5)/1.251(5); 1.237(5)/1.270(5); 1.246(5)/1.233(5)
$(Hpyr^+)_2Htma^{2-}\cdot$bipy$\cdot H_2O$	1.269(4)/1.229(5); 1.244(5)/1.249(5)
$(Htmp^+)_2Htma^{2-}\cdot$1.5ebipy$\cdot 3H_2O$	1.267(3)/1.252(3); 1.253(3)/1.256(3)
$(Htmp^+)_2Htma^{2-}\cdot$phpy$\cdot$0.5ebipy$\cdot 4H_2O$	1.254(7)/1.261(7); 1.261(8)/1.245(8)
$Hpyr^+Htmp^+Htma^{2-}\cdot$bipy$\cdot H_2O$	1.258(4)/1.255(4); 1.265(4)/1.253(4)
$Hpyr^+H_2tma^-\cdot$phpy$\cdot$pbipy	1.252(3)/1.249(3)

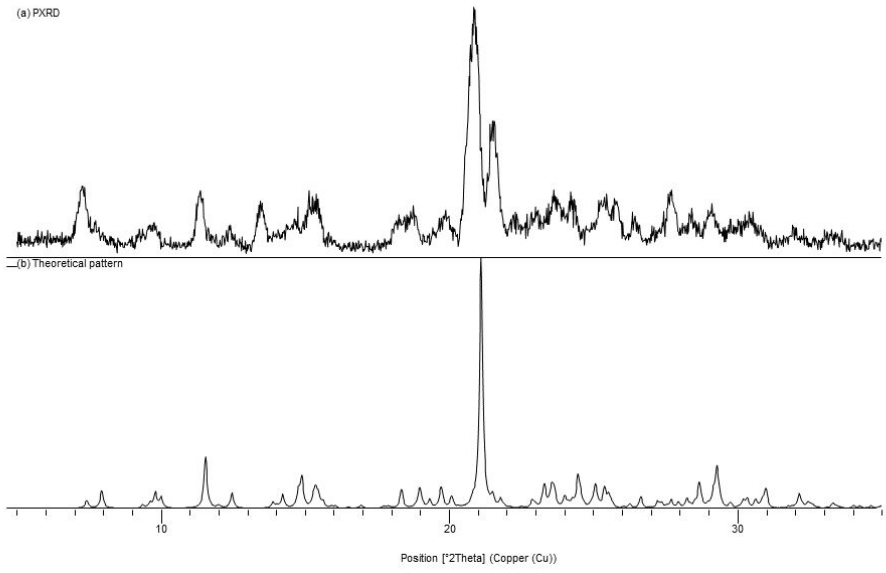

Figure S1. XRPD pattern of a 2:1 mixture of pyr and H_3tma milled for 30 min at room temperature in the presence of traces of methanol/water (top) and the theoretical XRPD pattern of the $(Hpyr^+)_2Htma^{2-}\cdot 3H_2O$ cocrystal calculated from the single crystal data (bottom).

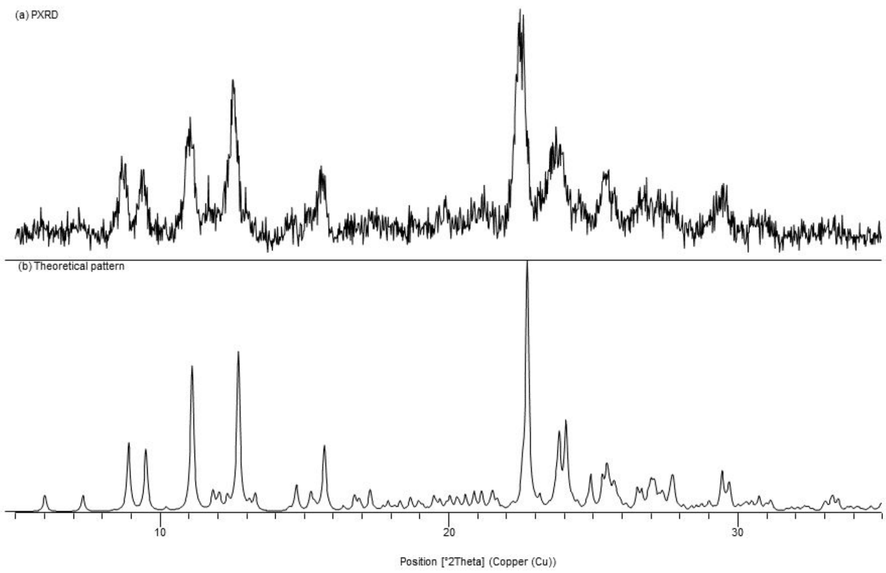

Figure S2. XRPD pattern of a 3:1 mixture of pyr and H_3tma milled for 30 min at room temperature in the presence of traces of methanol/water/acetonitrile (top) and the theoretical XRPD pattern of the $(Hpyr^+)_3tma^{3-}\cdot CH_3CN\cdot CH_3OH\cdot 2.5H_2O$ cocrystal calculated from the single crystal data (bottom).

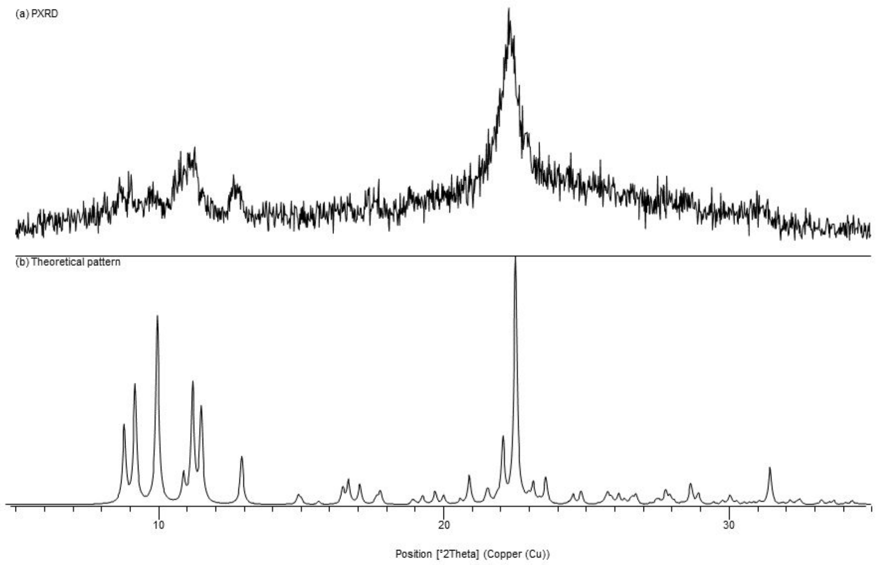

Figure S3. XRPD patterns of a 1:1 mixture of pyr and H_3tma milled for 30 min at room temperature in the presence of traces of methanol (top) and the theoretical XRPD pattern of the $Hpyr^+H_2tma^-{\cdot}H_2O$ cocrystal calculated from the single crystal data (bottom).

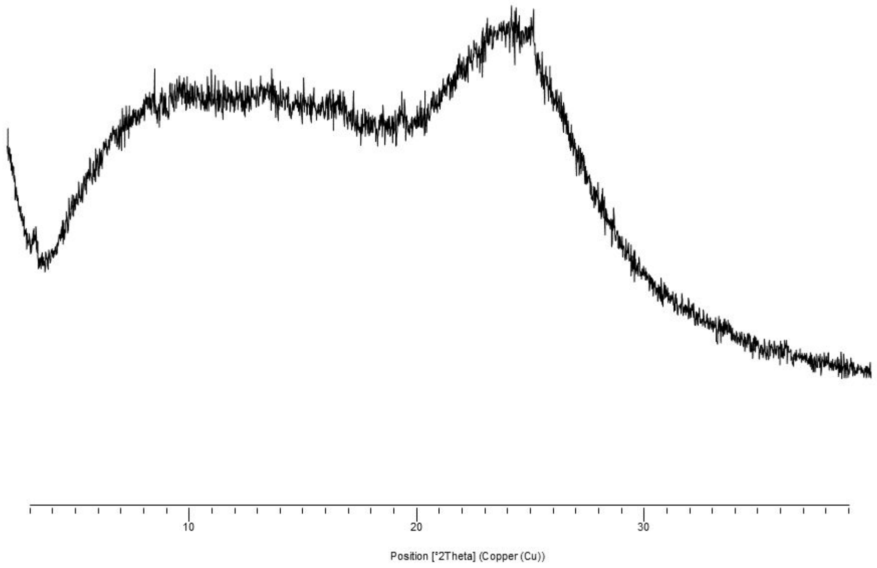

Figure S4. XRPD patterns of a 1:1 mixture of tmp and H_3tma milled for 30 min at room temperature

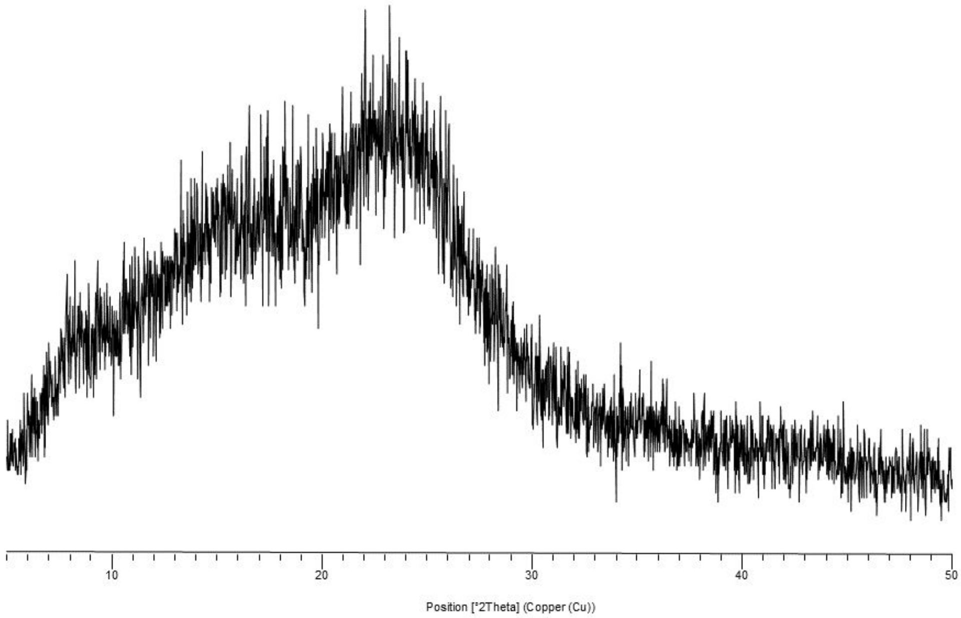

Figure S5. XRPD patterns of a 2:1 mixture of tmp and H_3tma milled for 30 min at room temperature

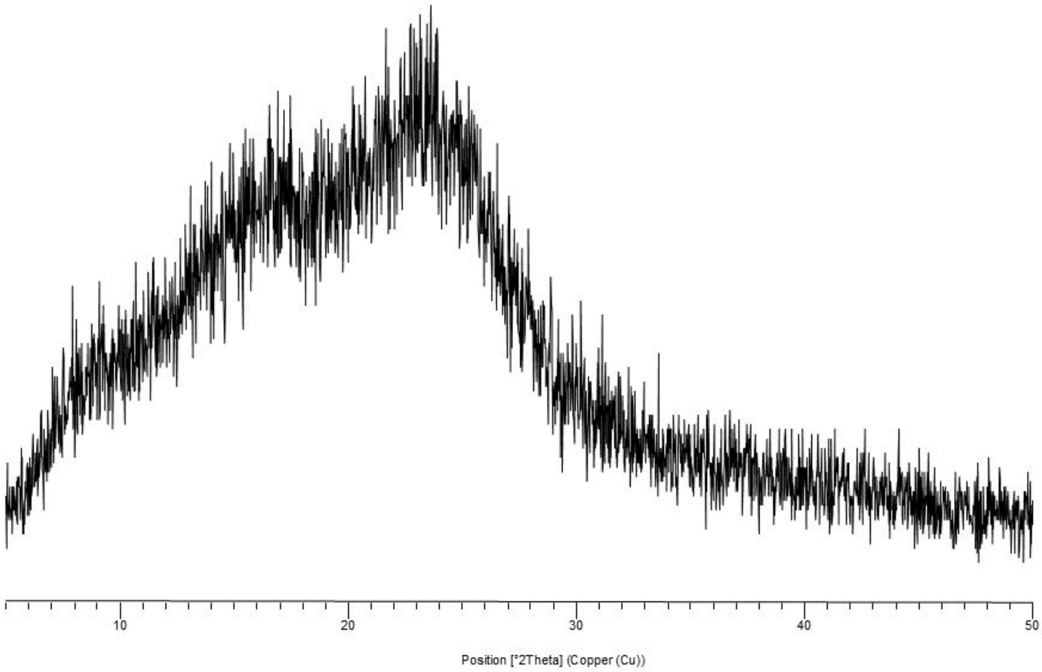

Figure S6. XRPD patterns of a 3:1 mixture of tmp and H_3tma milled for 30 min at room temperature

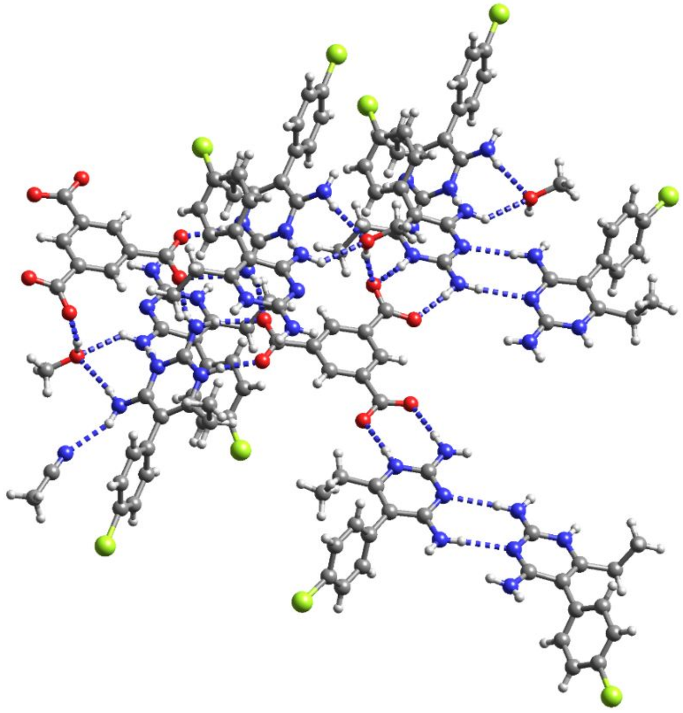

Figure S7. Crystal structure of $(Hpyr^{+})_3tma^{3-}\cdot CH_3CN\cdot CH_3OH\cdot 2.5H_2O$. The water molecule of crystallization is omitted for clarity.

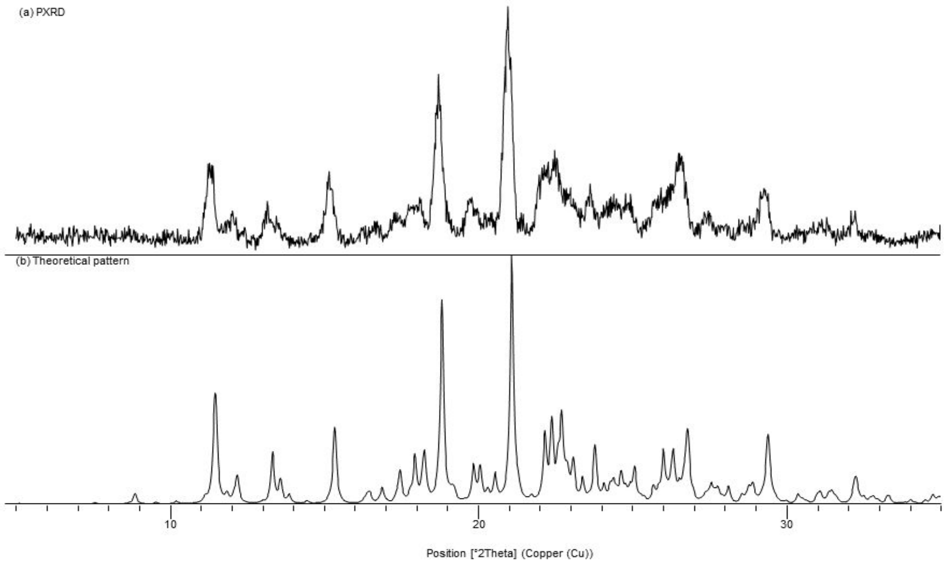

Figure S8. XRPD pattern of a 2:1:1 mixture of pyr, H_3tma and bipy after milling for 30 min in the presence of traces of methanol and water (top) and the theoretical XRPD pattern of $(Hpyr^{+})_2Hma^{2-}\cdot bipy\cdot H_2O$ calculated from the single crystal data (bottom).

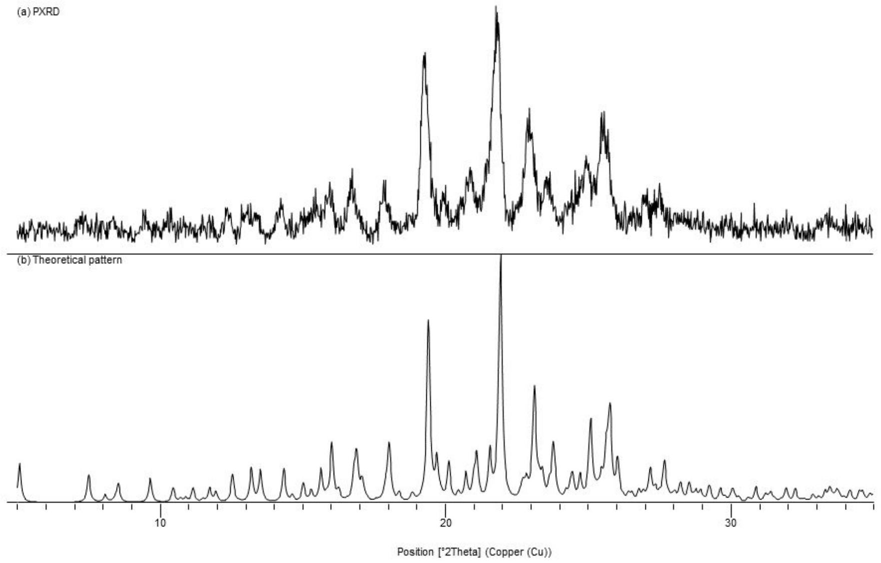

Figure S9. XRPD pattern of a 2:1:1 mixture of pyr, H_3tma and ebipy after milling for 30 min in the presence of traces of water and methanol (top) and the theoretical XRPD pattern of $(Hpyr^+)_2Htma^{2-}$ $\cdot$ebipy$\cdot H_2O\cdot CH_3OH$ calculated from the single crystal data (bottom).

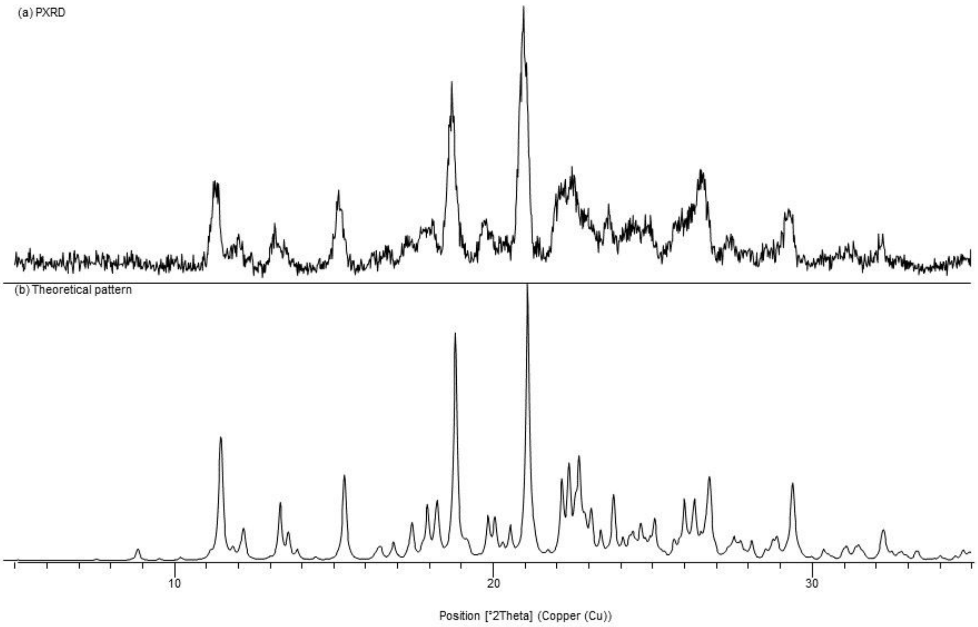

Figure S10. XRPD pattern of a 1:1:1.5 mixture of tmp, H_3tma and bipy after milling for 30 min in the presence of traces of water and methanol (top) and the theoretical XRPD pattern of $Htmp^+H_2tma^-$·1.5bipy·H_2O calculated from the single crystal data (bottom).

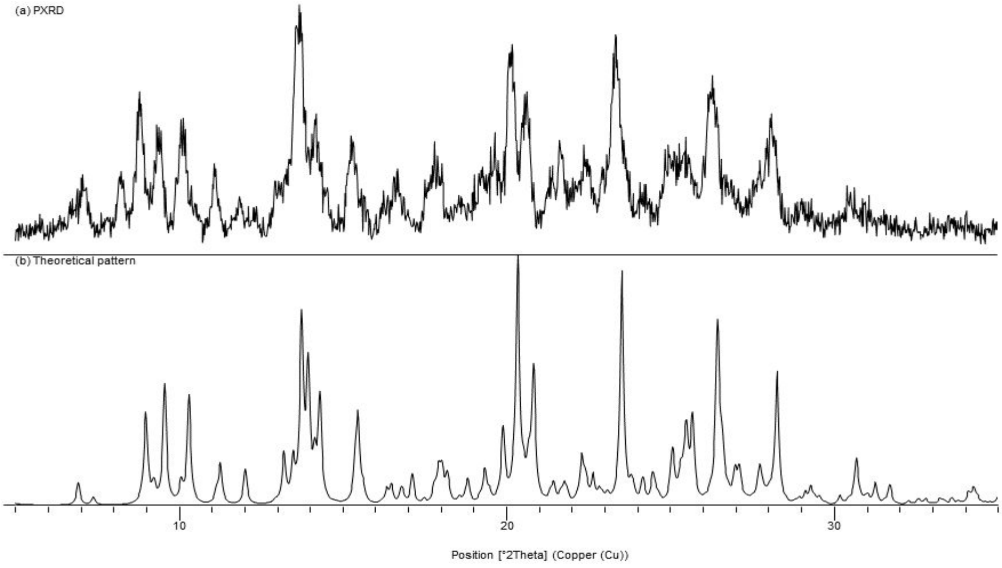

Figure S11. XRPD pattern of a 2:1:1.5 mixture of tmp, H_3tma and bipy after milling for 30 min in the presence of traces of water (top) and the theoretical XRPD pattern of $(Htmp^+)_2Htma^{2-}$·1.5bipy·$4H_2O$ calculated from the single crystal data (bottom).

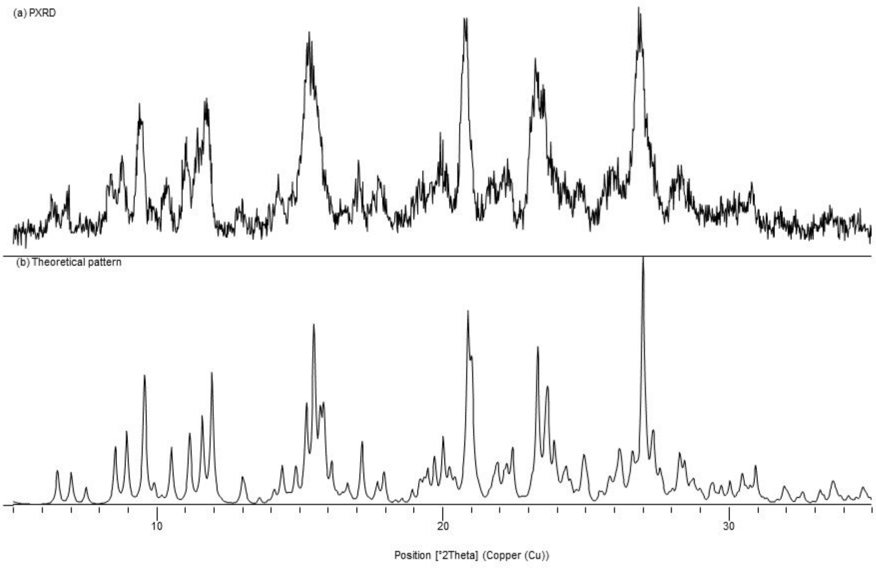

Figure S12. XRPD pattern of a 2:1:1.5 mixture of tmp, H_3tma and ebipy after milling for 30 min in the presence of traces of methanol and water (top) and the theoretical XRPD pattern of $(Htmp^+)_2Htma^{2-}$ ·1.5ebipy·$3H_2O$ calculated from the single crystal data (bottom).

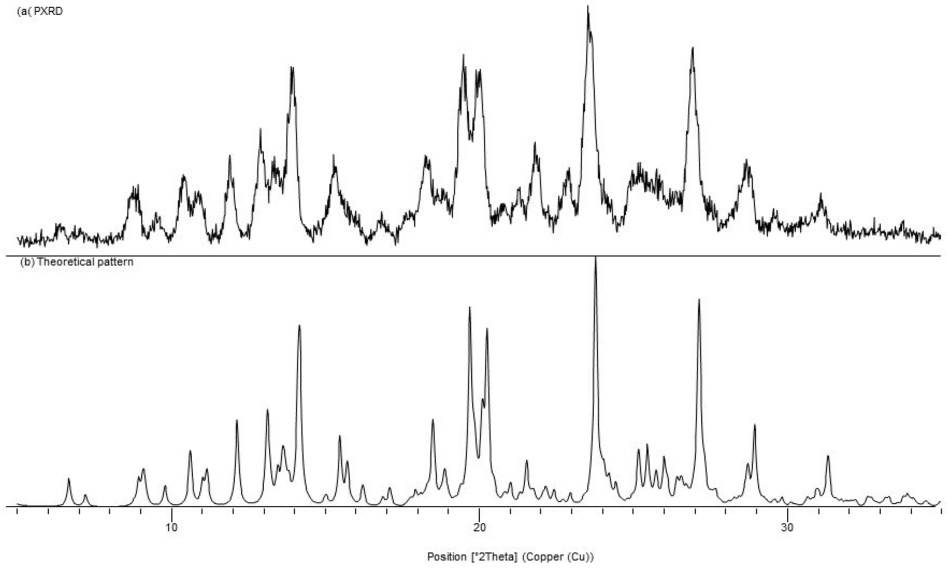

Figure S13. XRPD pattern of a 3:1:1 mixture of tmp, H_3tma and pbipy after milling for 30 min in the presence of traces of water (top) and the theoretical XRPD pattern of $(Htmp^+)_3tma^{3-}$·pbipy·$7H_2O$ calculated from the single crystal data (bottom).

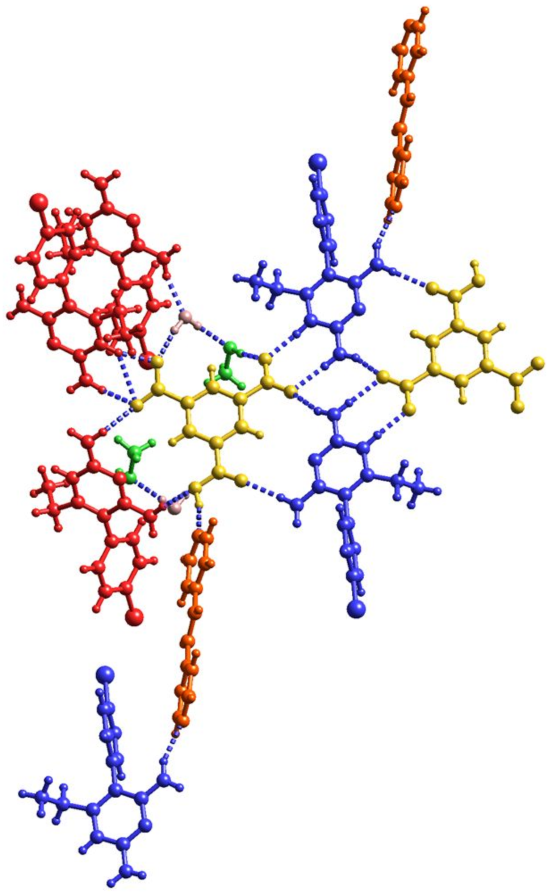

Figure S14. Crystal structure of $(Hpyr^+)_2Htma^{2-}$·ebipy·H_2O·CH_3OH. Only one conformer of the disordered ethylene group is shown. In addition to the H bonding described in the main text, there is H bonding between water of crystallization and carboxylate oxygen, between water molecules of crystallization and between water of crystallization and the C4-NH_2 group of one $Hpyr^+$ as well as between methanol and carboxylate oxygen and between methanol and water.

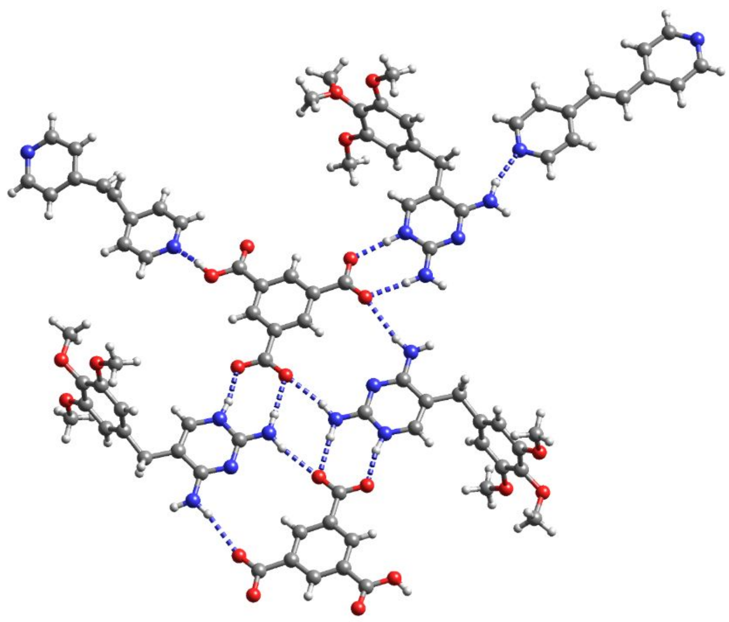

Figure S15. Crystal structure of $(Htmp^{+})_2Htma^{2-}\cdot 1.5ebipy\cdot 3H_2O$. Water and methanol molecules are omitted for clarity. Only one conformer of the disordered ethylene group is shown.

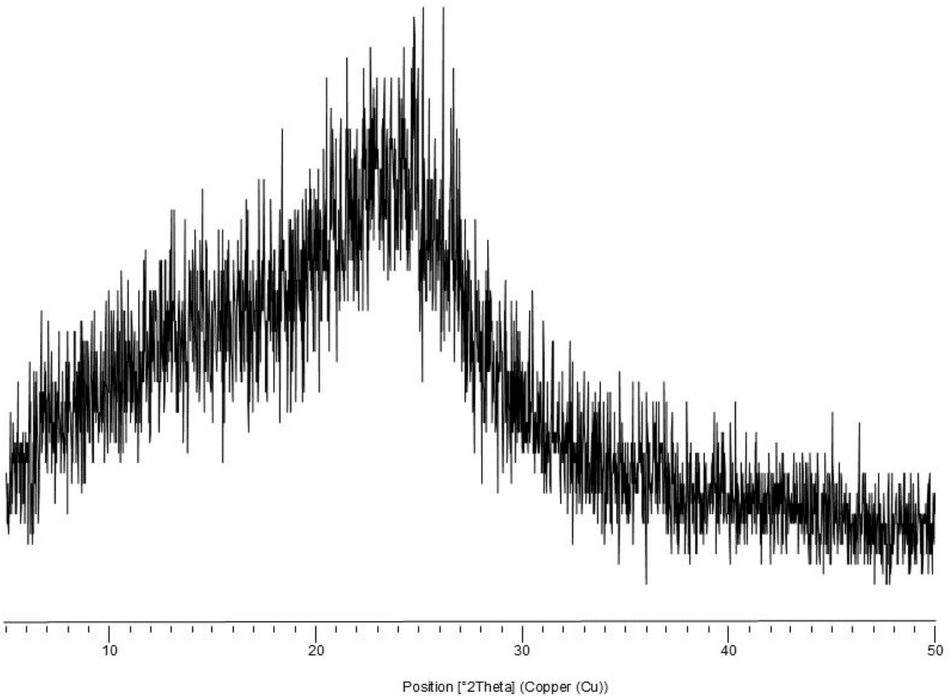

Figure S16. XRPD pattern of a 1:1:1 mixture of pyr, tmp, and H_3tma after milling for 30 min in the presence of traces of methanol.

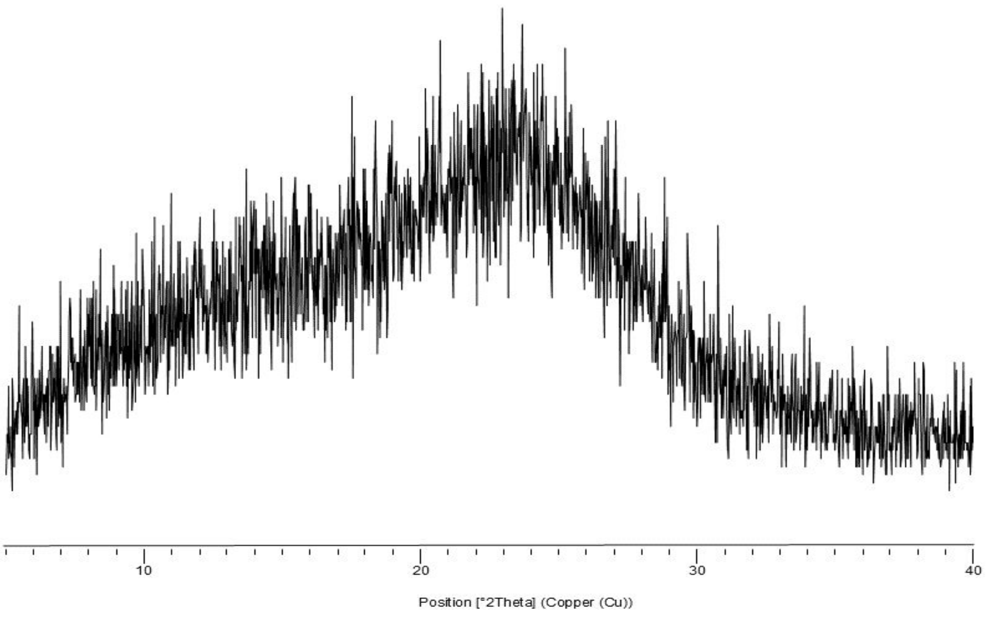

Figure S17. XRPD pattern of a 2:1:1 mixture of pyr, tmp, and H_3tma after milling for 30 min in the presence of traces of methanol and water.

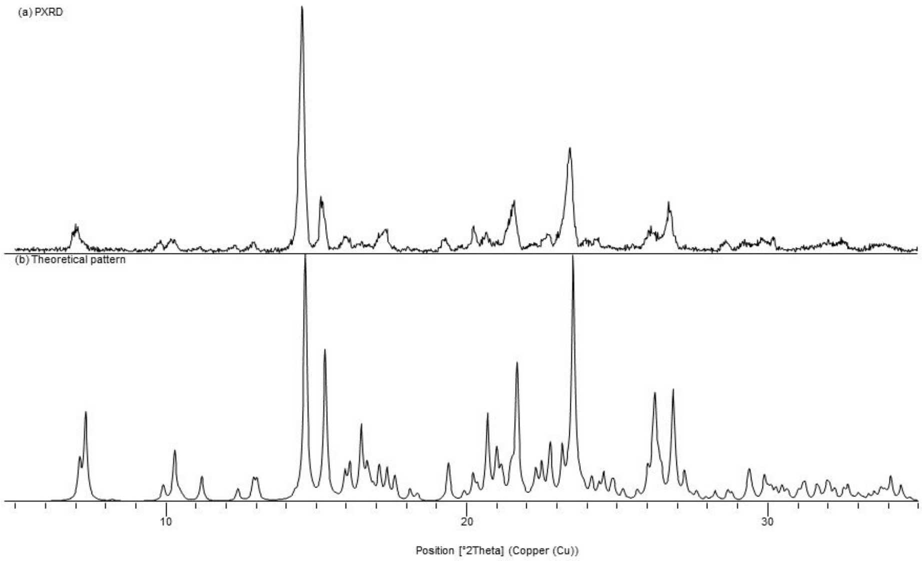

Figure S18. XRPD pattern of the isolated sample of $Hpyr^{+}Htmp^{+}Htma^{2-}$ (top) and the theoretical pattern calculated from the single crystal data (bottom).

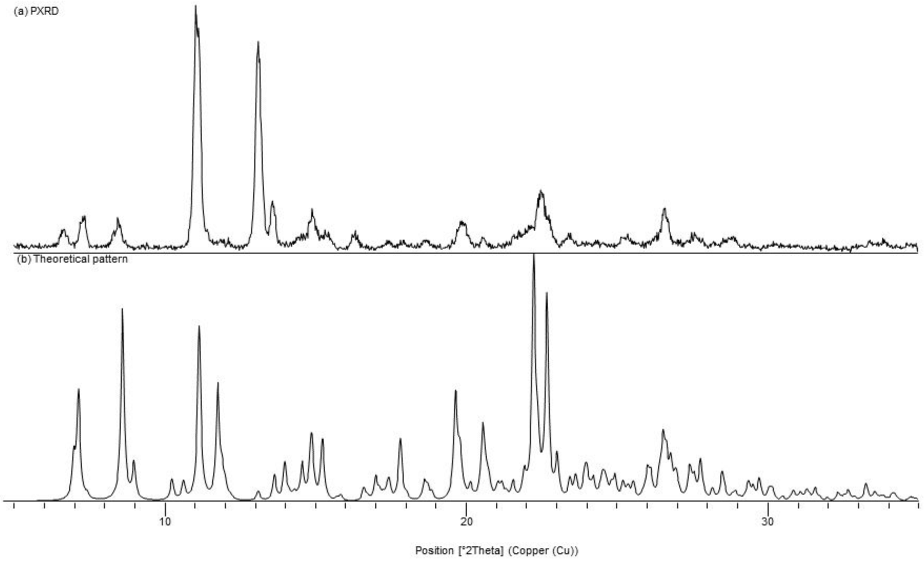

Figure S19. XRPD pattern of the isolated sample of $(Hpyr^{+})_2Htmp^{+}tma^{3-}{\cdot}2CH_3OH{\cdot}2H_2O^{-}$ (top) and the theoretical pattern calculated from the single crystal data (bottom).

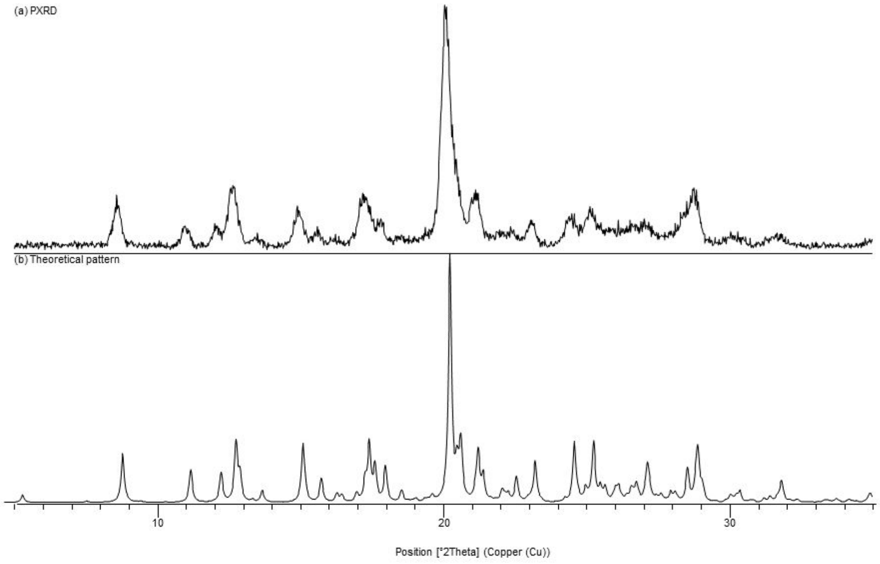

Figure S20. XRPD pattern of a 1:1:1:1 mixture of tmp, H_3tma, phpy and pbipy after milling for 30 min in the presence of traces of methanol and water (top) and the theoretical XRPD pattern of Hpyr$^+$H$_2$tma$^-$·phpy·pbipy calculated from the single crystal data (bottom).

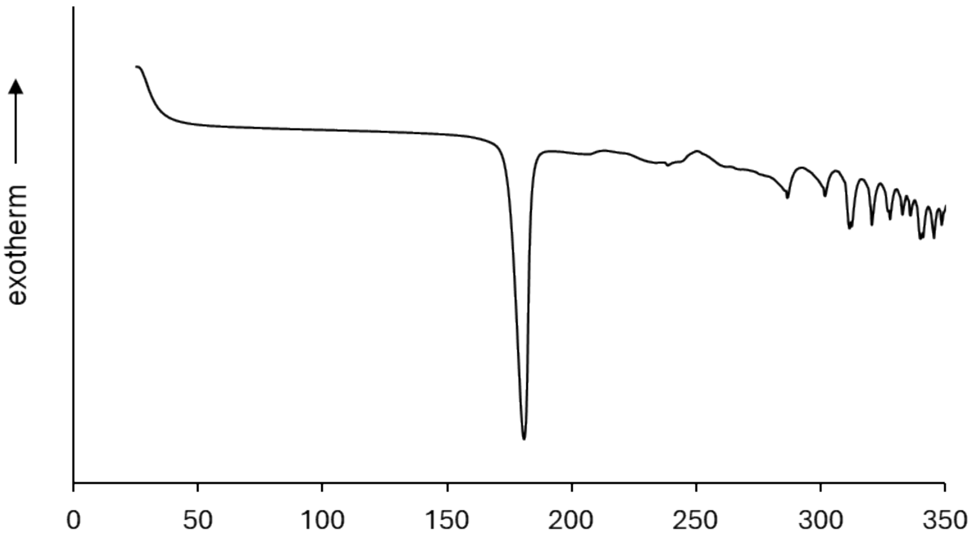

Figure S21. DSC plot of Hpyr$^+$H$_2$tma$^-$·phpy·pbipy prepared by ball-milling.

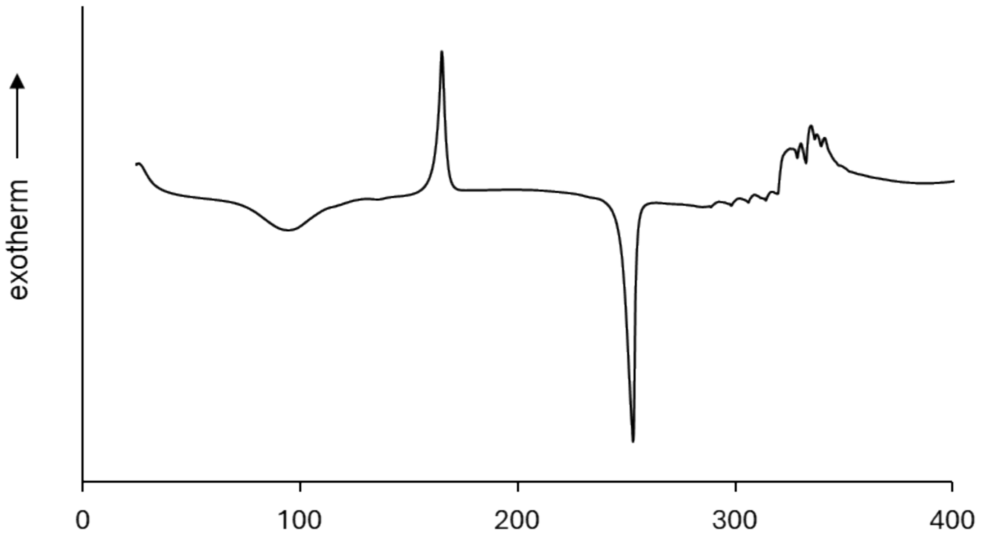

Figure S22. DSC plot of $Hpyr^{+}Htmp^{+}Htma^{2-}$ prepared by ball-milling. The broad endotherm around 100 °C is probably due to the evaporation of surface water.

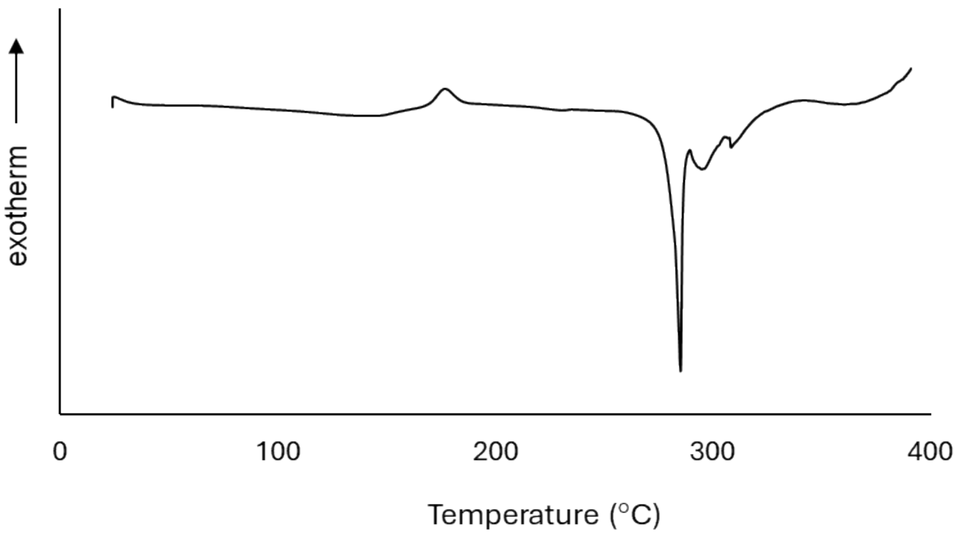

Figure S23. DSC plot of $Htmp^{+}H_2tma^{-}\cdot 3H_2O$ prepared by milling.

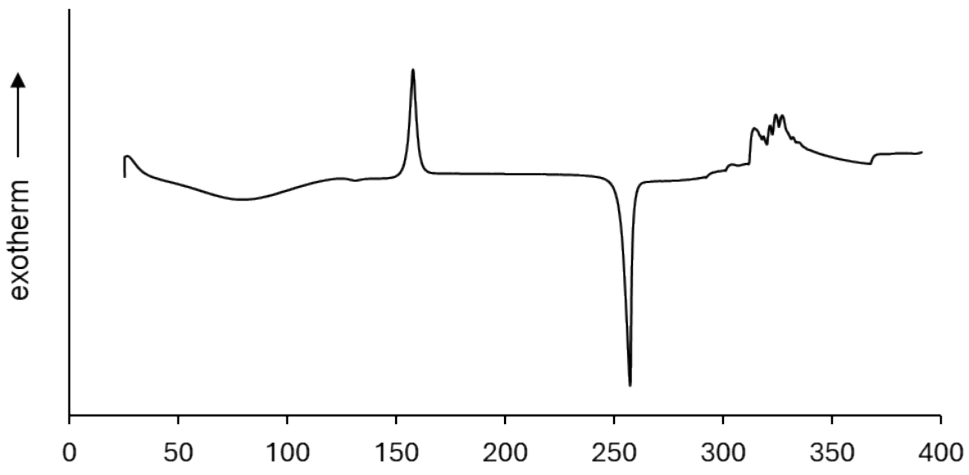

Figure S24. DSC plot of $(Htmp^{+})_2Htma^{2-}\cdot5H_2O$ prepared by ball-milling.

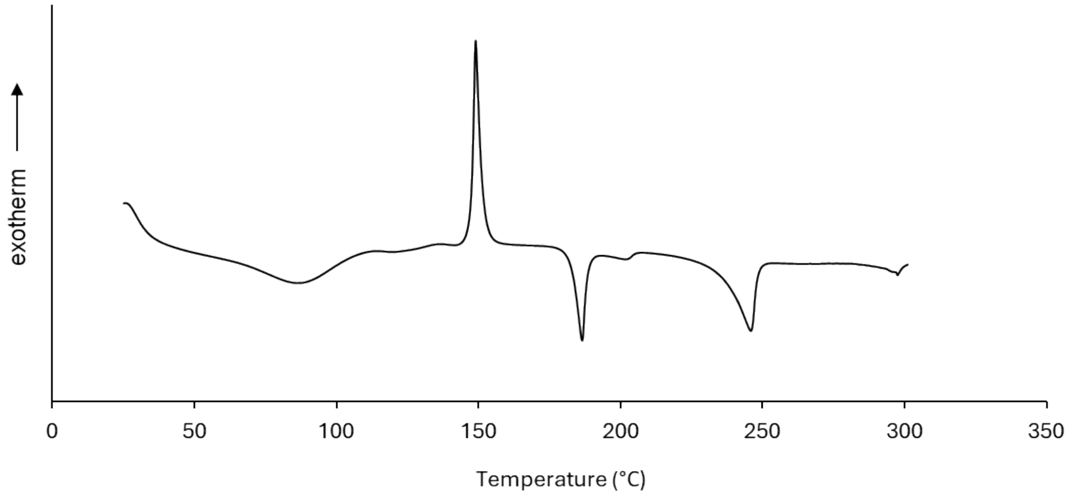

Figure S25. DSC plot of the amorphous sample obtained after milling a 2:1:1 mixture of pyr, tmp and H_3tma in the presence of traces of methanol and water.

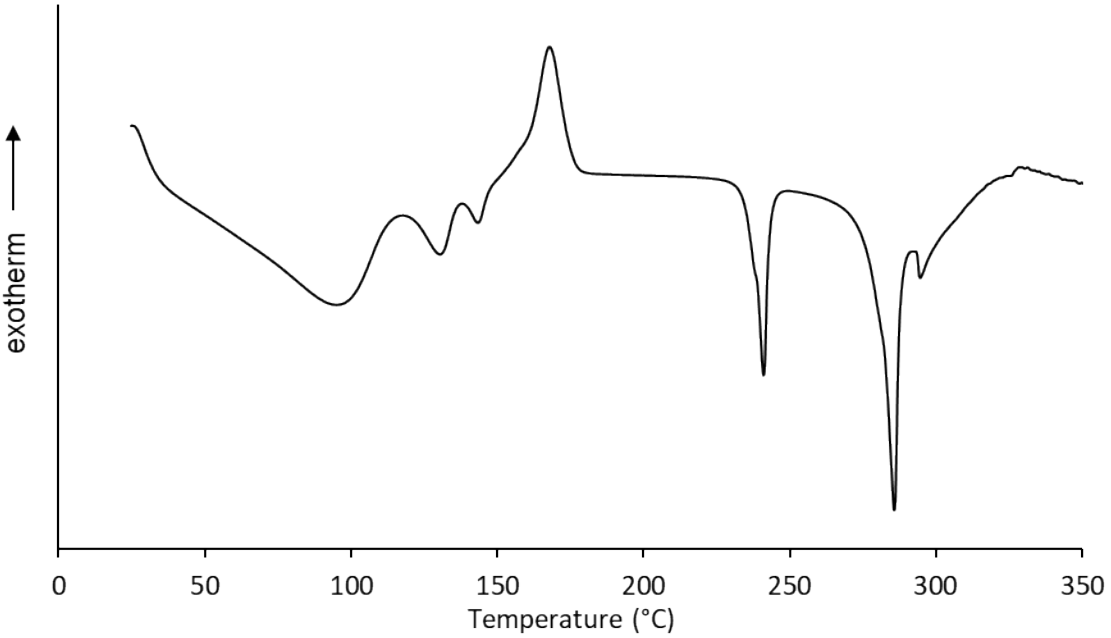

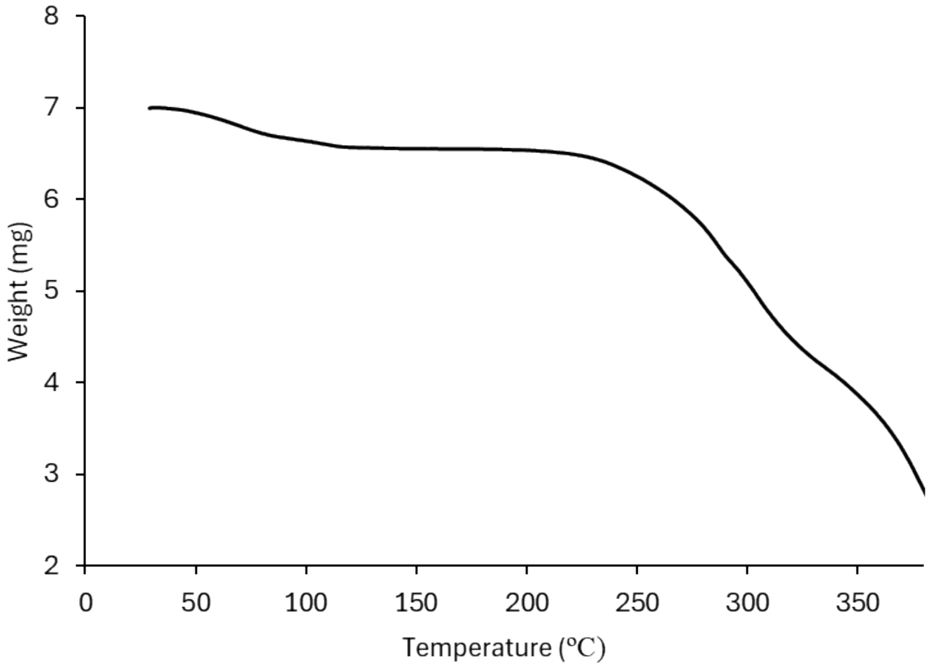

Figure S26. DSC (top) and TGA (bottom) plots of $(Hpyr^+)_3tma^{3-}\cdot CH_3CN\cdot CH_3OH\cdot 2.5H_2O$ prepared by milling.

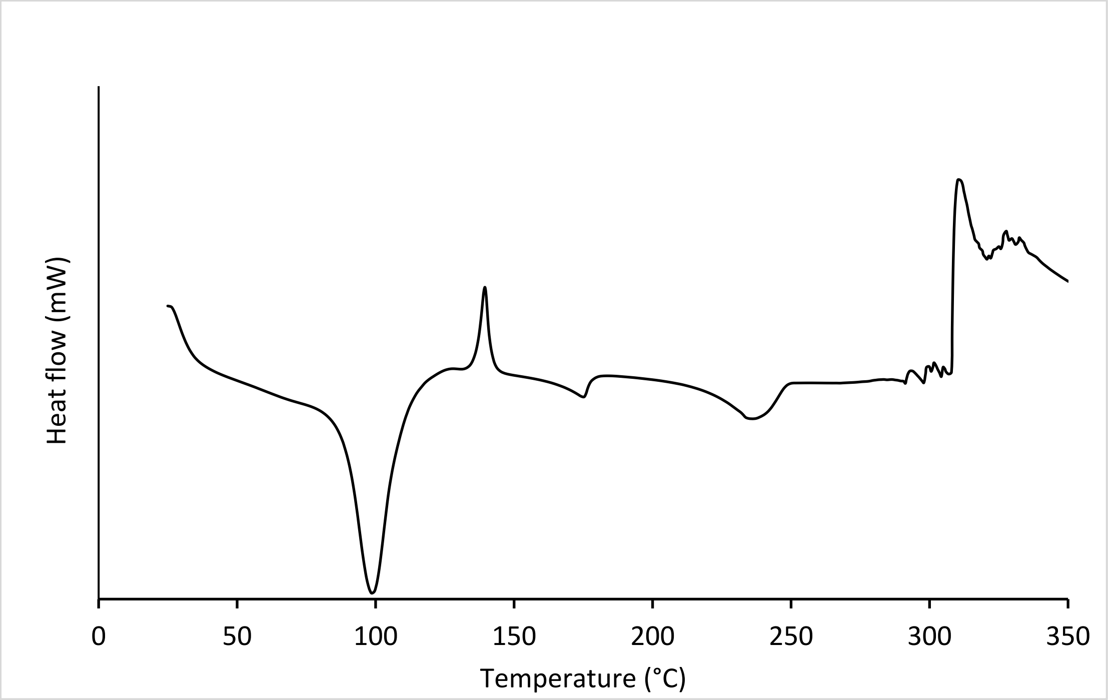

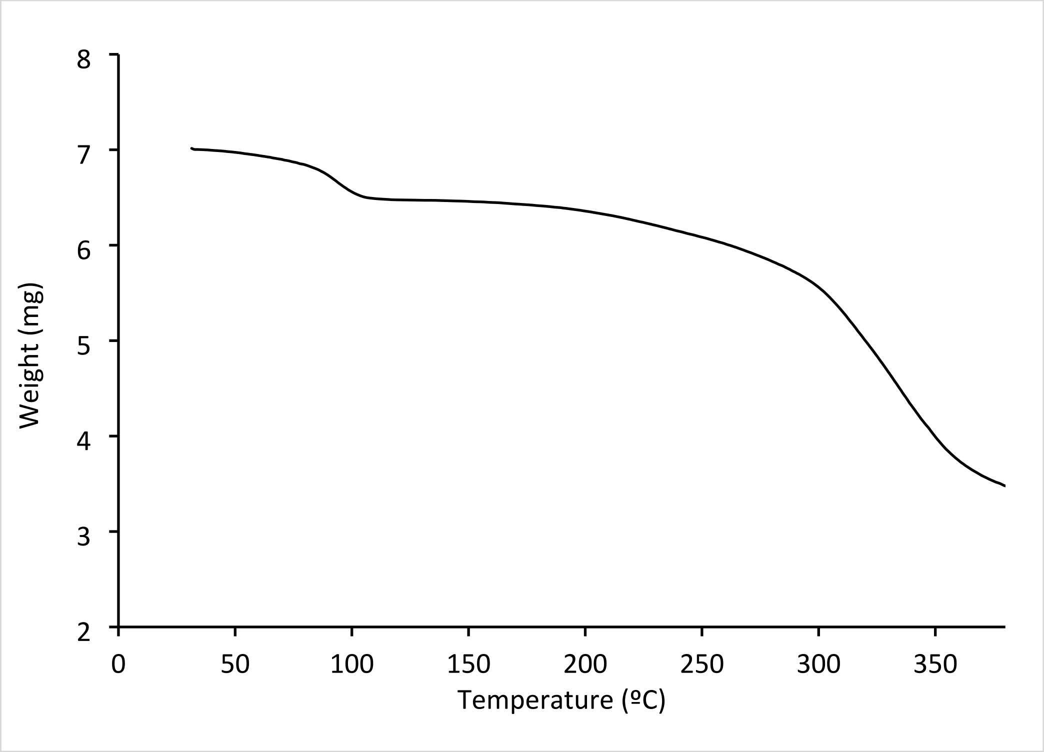

Figure S27. DSC (top) and TGA (bottom) plots of $(Htmp^{+})_3tma^{3-}\cdot pbipy\cdot 7H_2O$ prepared by milling.

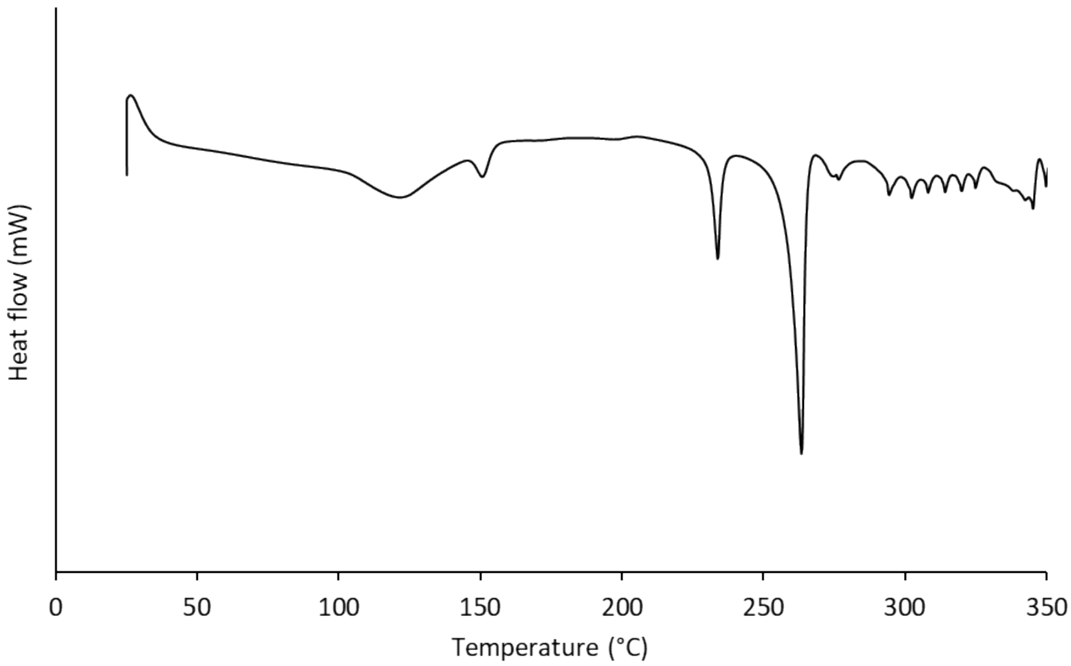

Figure S28. DSC plot of $(Hpyr^{+})_2Htma^{2-}$·ebipy·H_2O·CH_3OH prepared by milling.

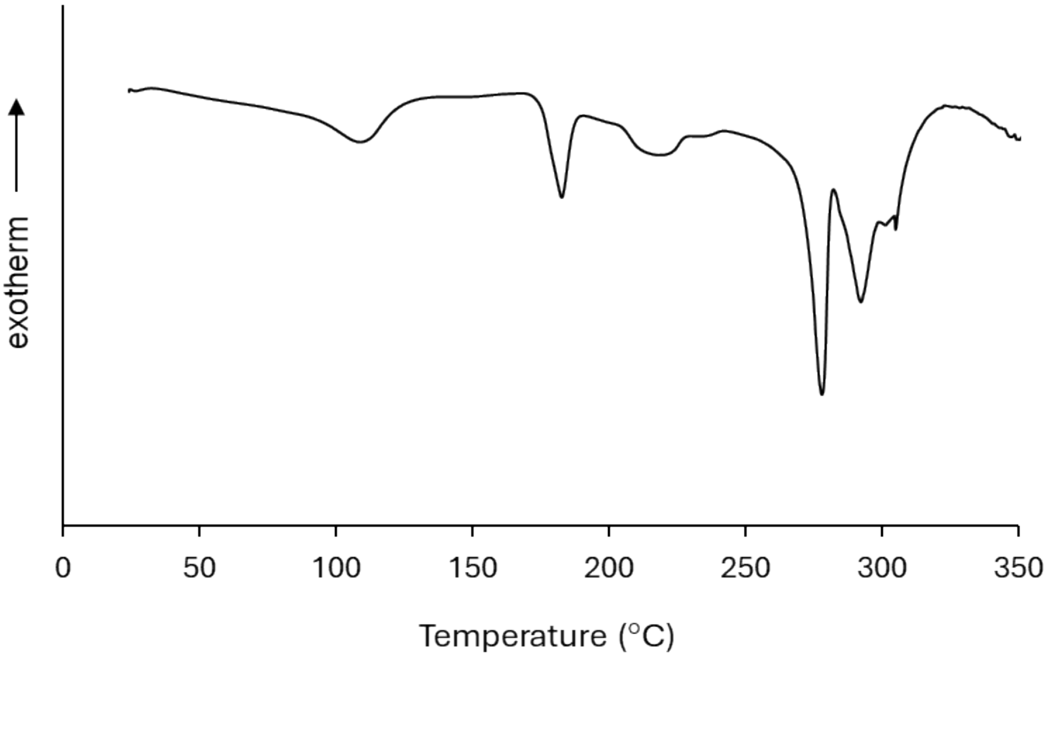

Figure S29. DSC plot of $(Hpyr^{+})_2Htma^{2-}$·bipy·H_2O prepared by milling.

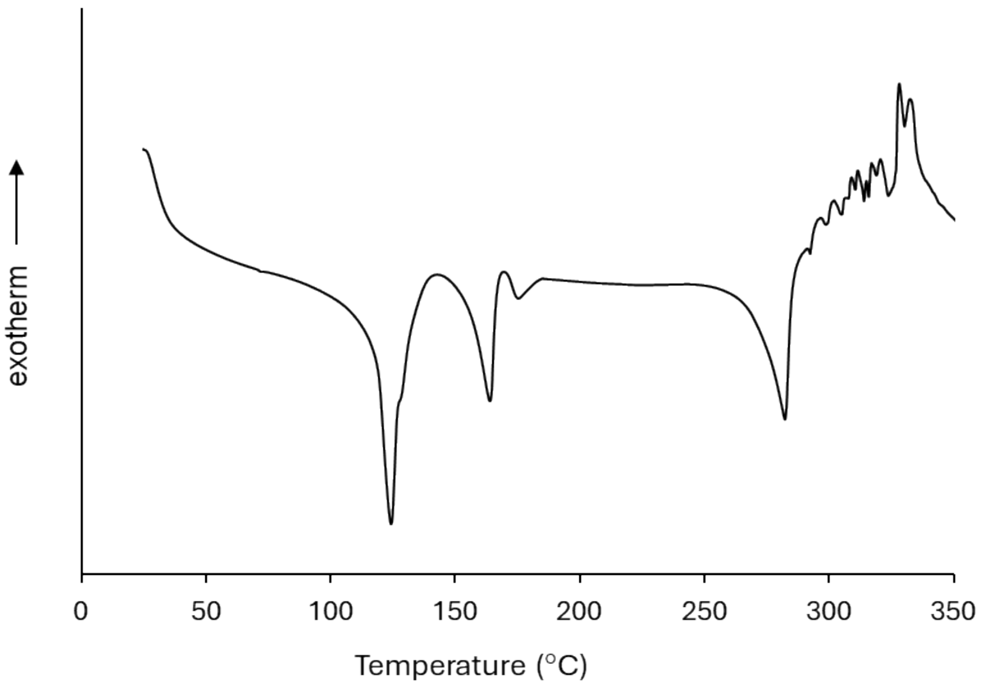

Figure S30. DSC plot of $(Htmp^+)_2Htma^{2-}$·phpy·0.5ebipy·$4H_2O$ prepared by milling.

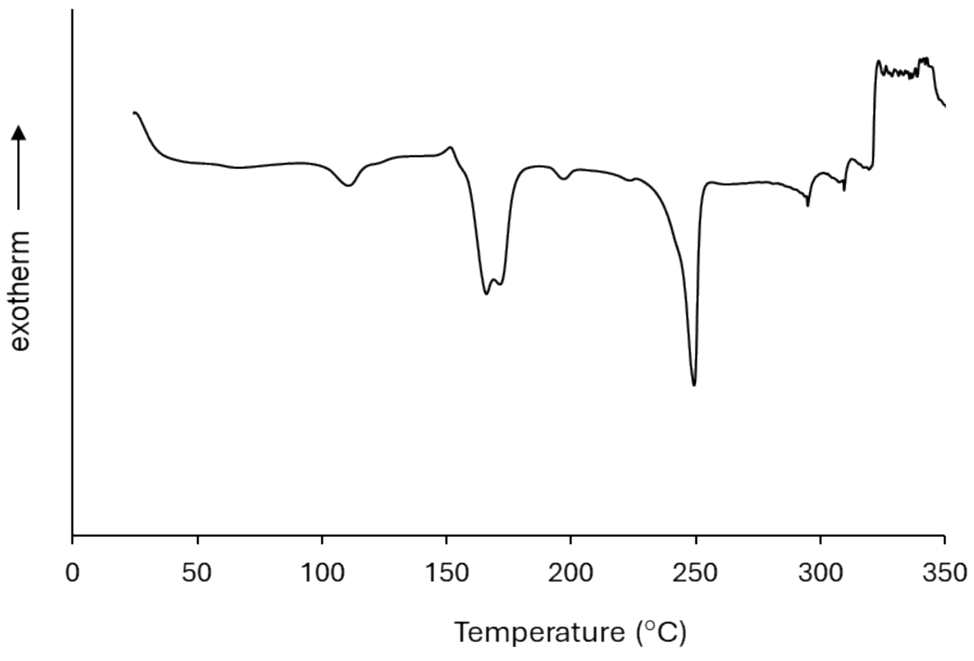

Figure S31. DSC plot of $Hpyr^+Htmp^+Htma^{2-}$·bipy·H_2O prepared by milling.

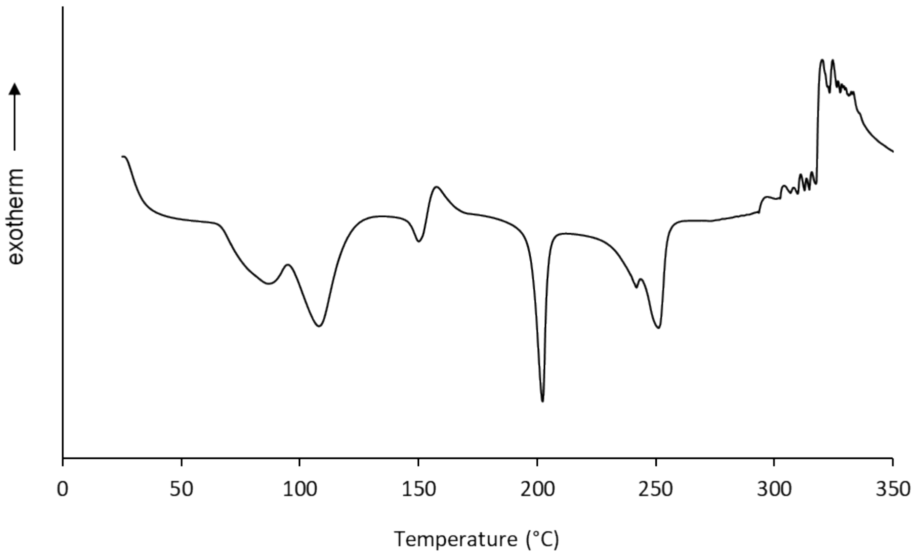

Figure S32. DSC plot of $(Htmp^{+})_2Htma^{2-}\cdot 1.5bipy\cdot 4H_2O$ prepared by milling.

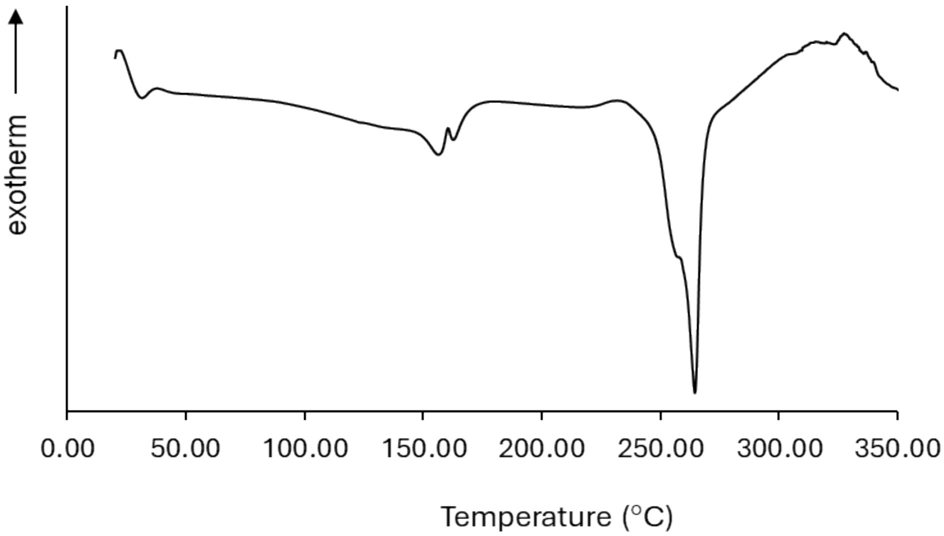

Figure S33. DSC plot of $Htmp^{+}H_2tma^{-}\cdot 1.5bipy\cdot H_2O$ prepared by milling.

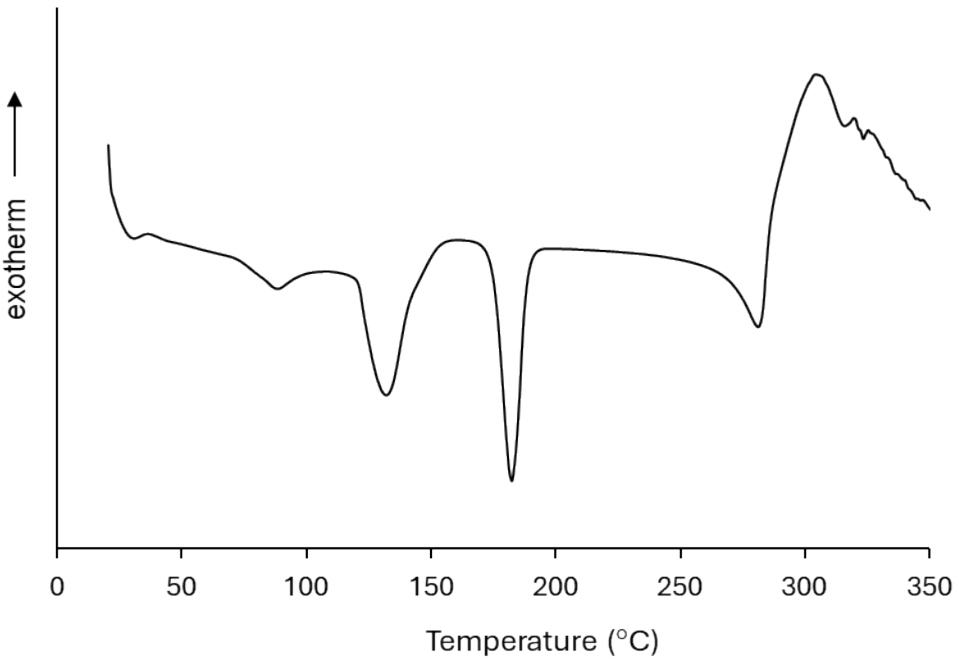

Figure S34. DSC plot of $(Htmp^+)_2Htma^{2-}{\cdot}1.5ebipy{\cdot}3H_2O$

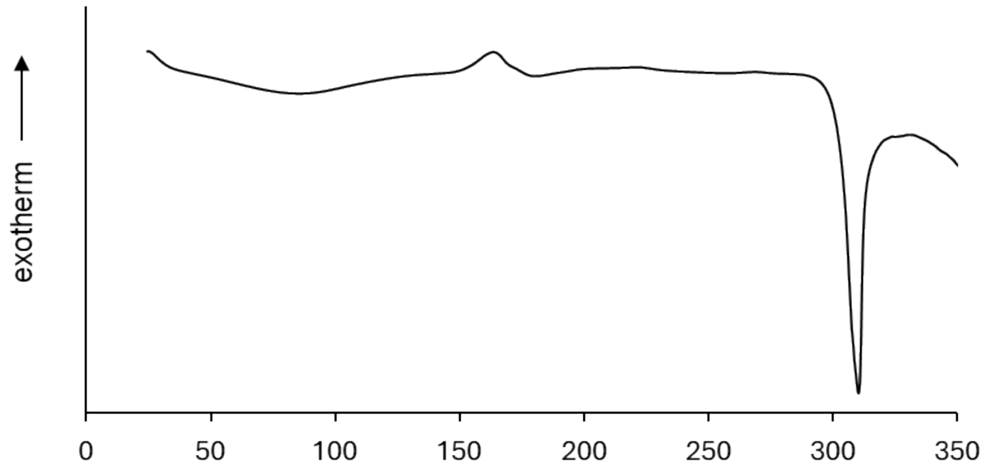

Figure S35. DSC plot of $Hpyr^+H_2tma^-{\cdot}H_2O$ prepared by ball-milling.
